# Supplementary material for: Targeted and high-throughput gene knockdown in diverse bacteria using synthetic sRNAs
Source: Nat Commun. 2023 Apr 24;14:2359. doi: 10.1038/s41467-023-38119-y (PMC10126203; doi:10.1038/s41467-023-38119-y)
Supplement: Supplementary file 1 — Supplementary Information [file 41467_2023_38119_MOESM1_ESM.pdf]

Targeted and high-throughput gene knockdown in diverse bacteria using synthetic sRNAs

Cho and Yang *et al.*

0

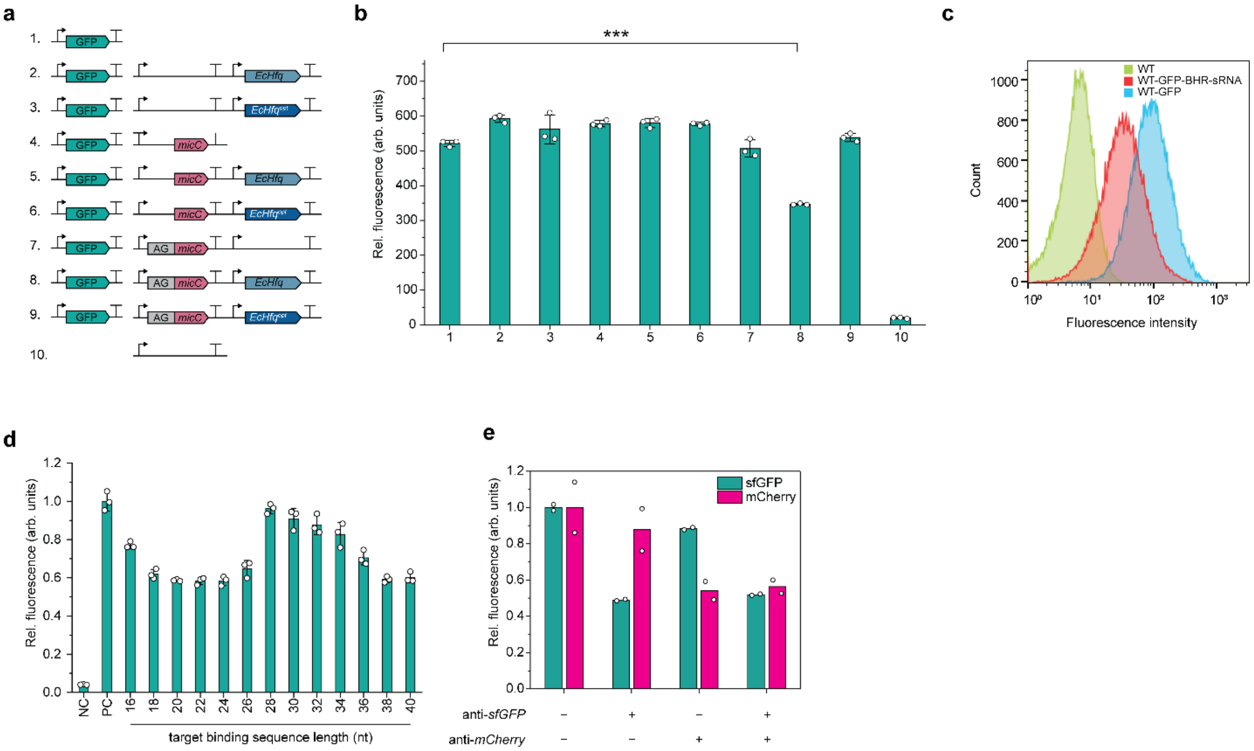

Supplementary Fig. 1. Construction and characterization of synthetic sRNA platforms in *C. glutamicum* ATCC 13032. a, Plasmid configurations for the reporter gene (*GFP*) and *E. coli*-based sRNA constructs. AG, anti-GFP. **b**, GFP fluorescence knockdown by various *E. coli*-based sRNA systems in *C. glutamicum*. The numbers in the x-axis correspond to the plasmid constructs in the panel a. The MicC-EcHfq construct (construct 8) resulted in the highest knockdown efficiency. ***$P < 0.001$, determined by two-tailed Student's *t*-test. Error bars, mean ± SD ($n = 3$ biologically independent samples). **c**, Fluorescent cell populations from the cultures of *C. glutamicum* strains. Representative histograms from three independent rounds of flow cytometry are shown, visualized with FlowJo. **d**, Effect of the length (nt) of target binding sequence on *sfGFP* repression efficiency. Abbreviations: NC, negative control; PC, positive control. Error bars, mean ± SD ($n = 3$ biologically independent samples). **e**, Orthogonal or simultaneous knockdown of the fluorescent reporter genes (encoding sfGFP and mCherry) by BHR-sRNA in *C. glutamicum*. $n = 2$ biologically independent samples. arb. units: arbitrary units. Source data are provided as a Source Data file.

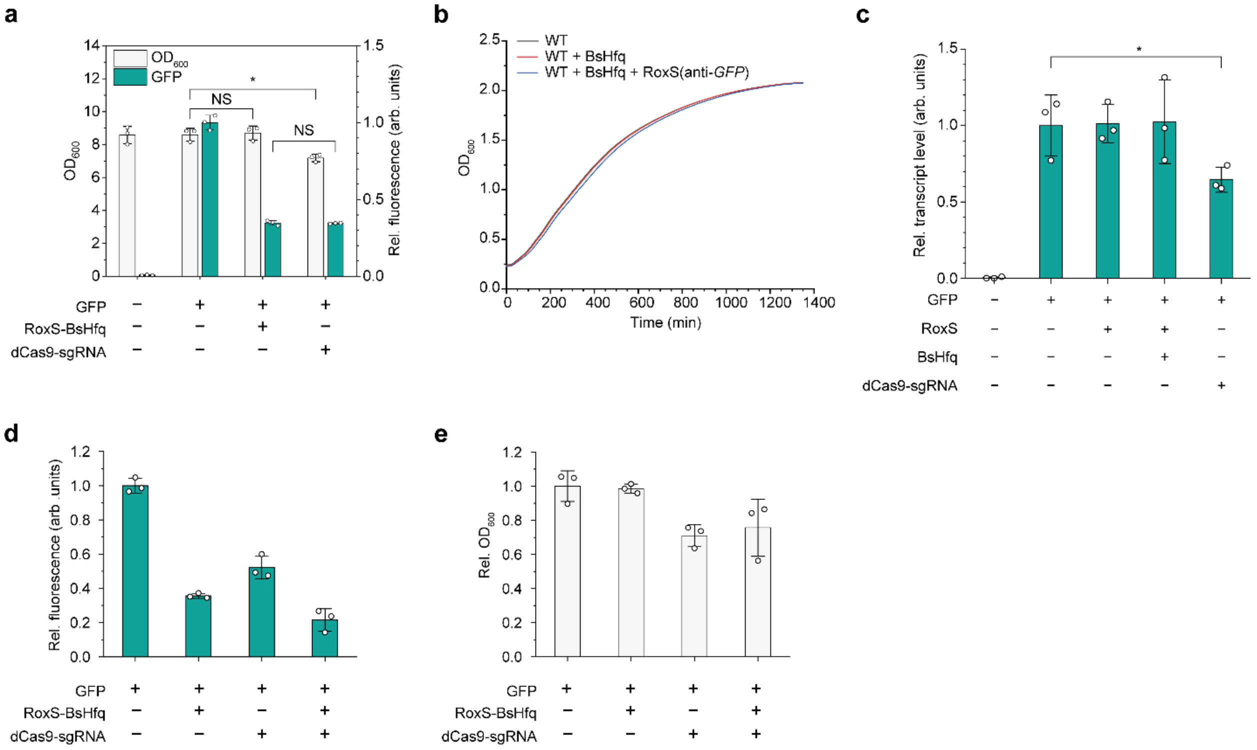

Supplementary Fig. 2. Comparison of the BHR-sRNA system with the CRISPRi system. a, Comparison of reporter (GFP) knockdown efficiency between BHR-sRNA and CRISPRi in *C. glutamicum*. Fluorescence measured with FACS (MoFlo XDP, Beckman Coulter). $*P < 0.025$, determined by two-tailed Student's *t*-test. *P*-value thresholds were adjusted using Bonferroni correction (corrected significance levels represented as α/m; α, original significance level; m, number of hypotheses). **b**, Growth profiles of *C. glutamicum* (WT), WT harboring BsHfq, and WT harboring the RoxS-BsHfq construct (anti-*GFP*) measured with Spark Multimode Microplate Reader (Tecan). Each curve represents a smooth line made by connecting the average OD_{600} values at each time point of triplicate experiments. **c**, Relative GFP transcript levels from *C. glutamicum* strains. Introduction of the anti-*GFP* sRNA constructs did not affect the transcript levels of GFP while introduction of the anti-*GFP* CRISPRi construct resulted in reduced GFP transcript level. $*P < 0.05$, determined by two-tailed Student's *t*-test. **d**, Comparison of reporter (GFP) knockdown efficiency among BHR-sRNA, CRISPRi, and dual-knockdown system combining both BHR-sRNA and CRISPRi. **e**, Relative maximum cell growth of the strains in panel **d**. (**d,e**) Fluorescence and OD_{600} measured with Spark Microplate Reader (Tecan). Abbreviations: NS, not significant; arb. units, arbitrary units. (**a**, **c-e**) Error bars, mean ± SD ($n = 3$ biologically independent samples). Source data are provided as a Source Data file.

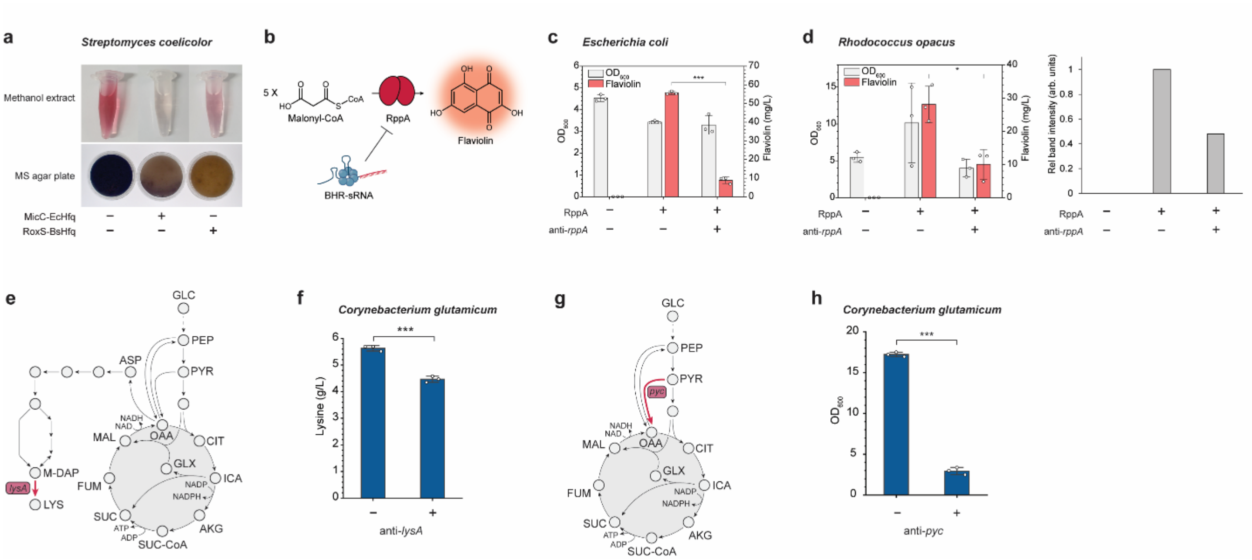

Supplementary Fig. 3. Knockdown of metabolic genes using the BHR-sRNA system result in alteration in phenotypes. **a**, Knockdown of *actIORFI* responsible for the production of a blue pigment actinorhodin in *S. coelicolor*. Plates were incubated at 30°C for one week before taking the photos. Actinorhodin was extracted from the dissolved MS agar plates (adjusted to pH ~2) by chloroform and resolubilized in methanol. **b**, Schematic representation of the inhibition of flaviolin production through the knockdown of *rppA* encoding a type III polyketide synthase by BHR-sRNA. THN, 1,3,6,8-tetrahydroxynaphthalene; spont., spontaneous. **c**, Knockdown of *rppA* responsible for flaviolin production by BHR-sRNA in *E. coli*. **d**, Knockdown of *rppA* by BHR-sRNA in *R. opacus*, with measured relative band intensities of RppA from SDS-PAGE. **e**, Metabolic pathways toward L-lysine production in *C. glutamicum* BE strain with the target *lysA* gene to be knocked down. **f**, Knockdown of *lysA* in a L-lysine overproducer *C. glutamicum* BE using BHR-sRNA results in reduced L-lysine production. **g**, Central metabolic pathways in *C. glutamicum* ATCC 13032 strain with the target *pyc* gene to be knocked down. **h**, Knockdown of *pyc* in *C. glutamicum* ATCC 13032 using BHR-sRNA results in reduced growth in a medium containing lactate as the sole carbon source. (**c,d,f,h**) Error bars, mean ± SD (n = 3 biologically independent samples). $*P < 0.05$, $**P < 0.01$, $***P < 0.001$, determined by two-tailed Student's t-test. Abbreviation: NS, not significant; arb. units, arbitrary units. Source data are provided as a Source Data file.

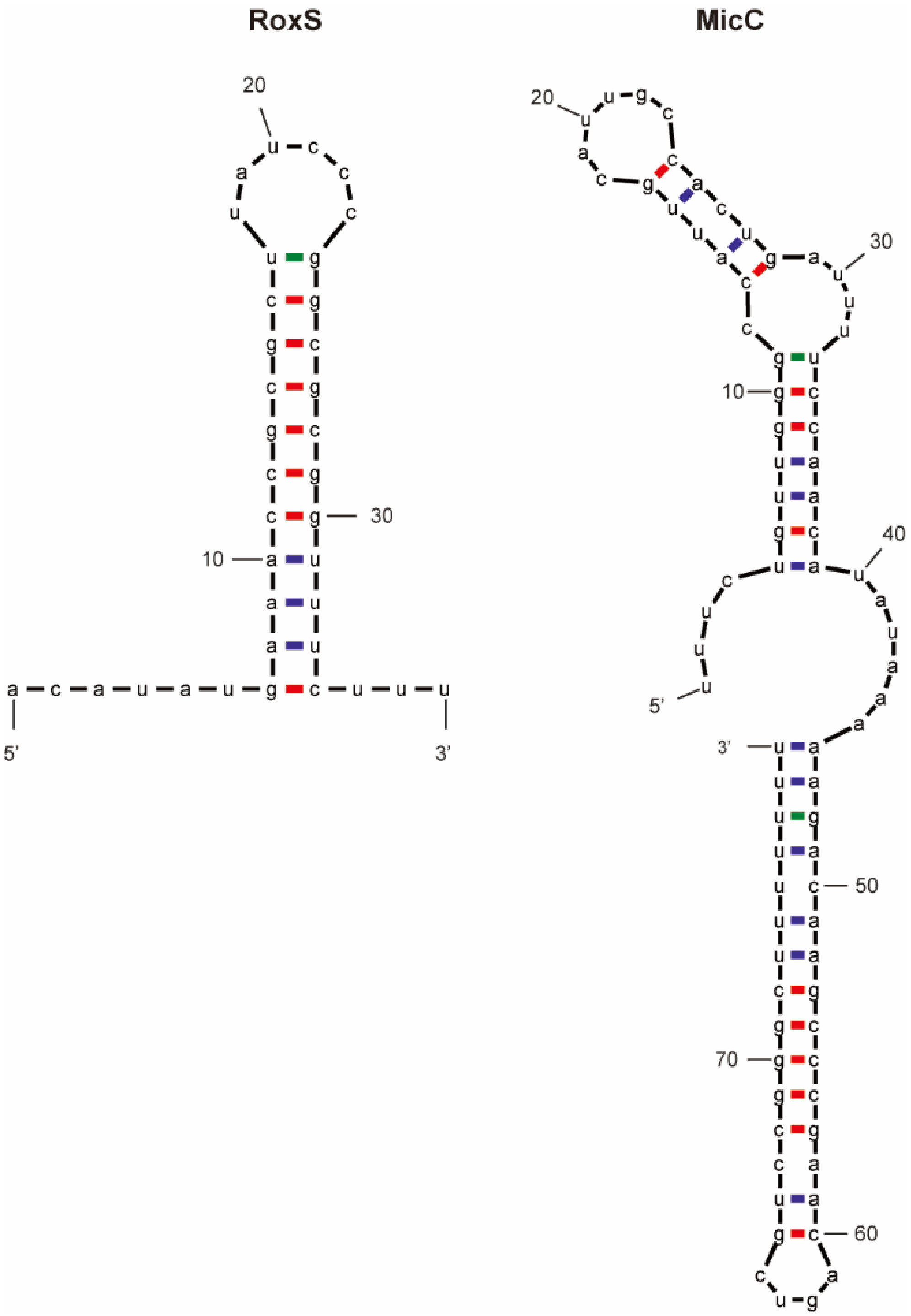

Supplementary Fig. 4. The predicted secondary structures of RoxS and MicC scaffolds. The predicted structures were generated using mFold RNA folding Form Version 2.3 at 30° C. The 3' stem loop structure of MicC corresponds to the terminator.

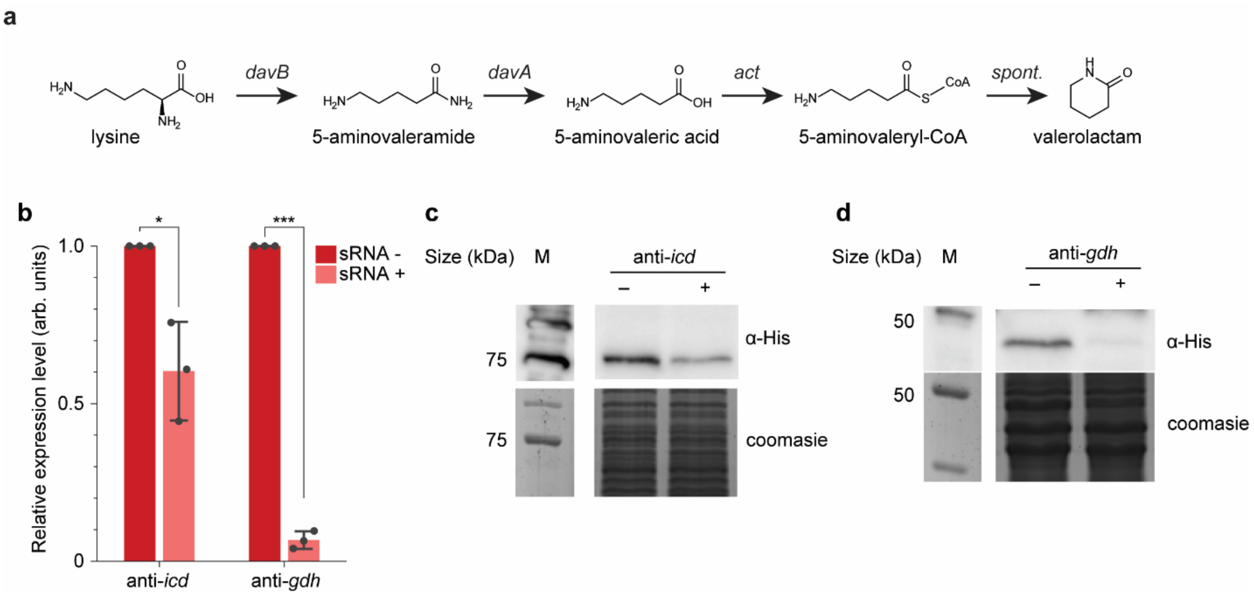

Supplementary Fig. 5. Biosynthesis of valerolactam in *C. glutamicum*. a, Biosynthetic pathway of valerolactam from L-lysine. **b**, Reduced production of target proteins by the VL-His strain after introduction of the target-specific sRNAs. Target protein levels were estimated by measuring the intensity of western-blot bands using the Image Lab software. The measured western-blot band intensities were normalized to the total intensity of the Coomassie-stained SDS-PAGE bands. Error bars, mean ± SD (n = 3 biologically independent samples). *$P < 0.05$, ***$P < 0.001$, determined by two-tailed Student's *t*-test. Representative western-blot and SDS-PAGE bands from triplicate experiments prepared from the VL-His strain harboring **c** anti-*icd* sRNA or **d** anti-*gdh* sRNA. Arrows denote the target protein bands. M, protein size marker. arb. units: arbitrary units. Source data are provided as a Source Data file.

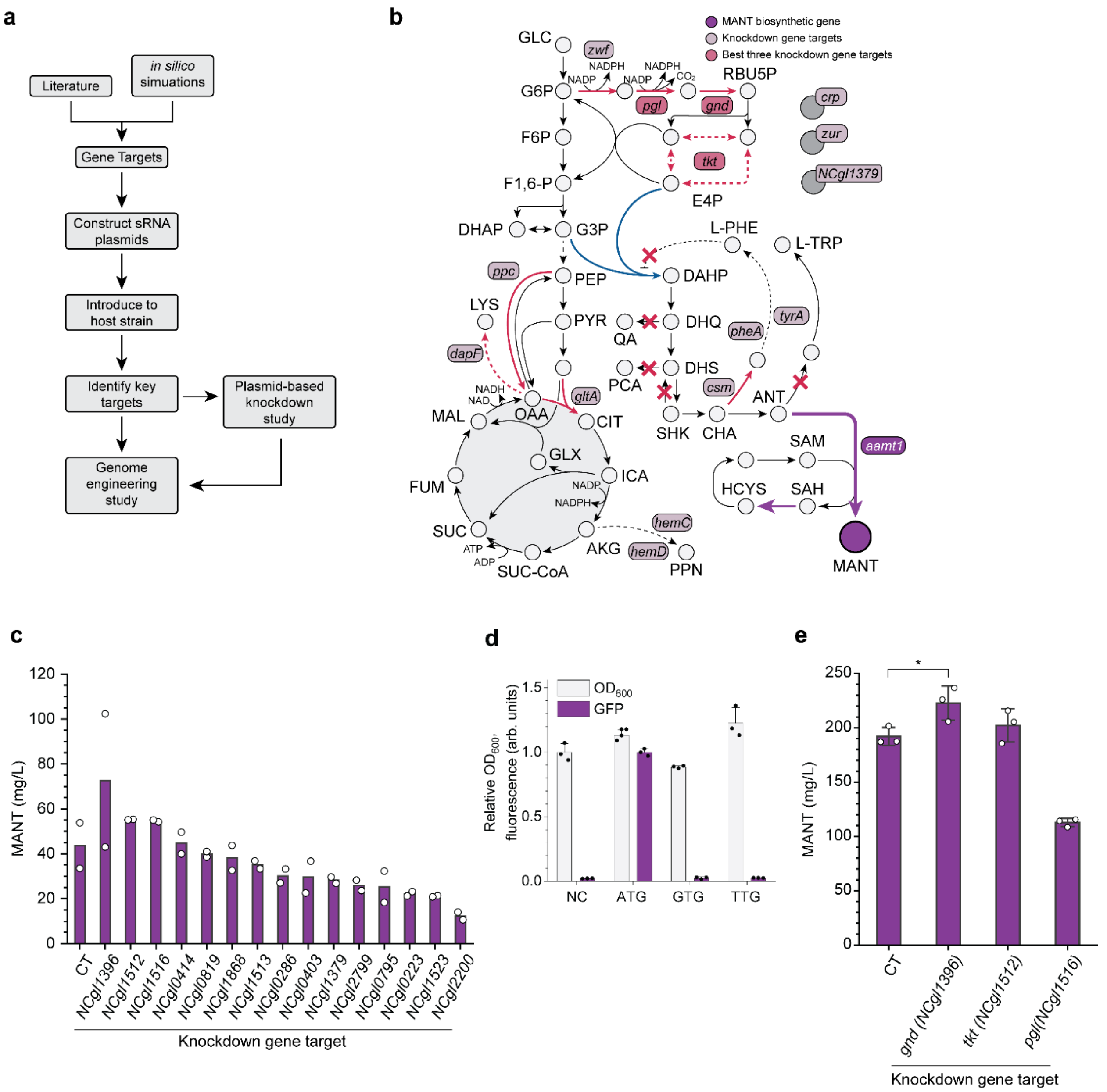

Supplementary Fig. 6. Rapid identification of gene knockdown targets for enhanced methyl anthranilate (MANT) production. a, Workflow to identify key target genes to be knocked down to enhance MANT production in *C. glutamicum*. **b**, Metabolic pathways for the biosynthesis of MANT, illustrating gene knockdown targets selected (light pink boxes) and experimentally validated target genes that showed enhanced MANT production (deep pink boxes). Abbreviations: GLC, glucose; G6P, glucose 6-phosphate; F6P, fructose 6-phosphate; RBU5P, ribulose; F1,6-P, fructose 1,6-bisphosphate; DHAP, dihydroxyacetonephosphate; G3P glyceraldehyde-3-phosphate; PEP, phosphoenolpyruvate; PYR, pyruvate; ; CIT, citrate; ICA, isocitrate; GLX, glyoxylate; AKG, α-ketoglutarate; PPN, protoporphyrin; SUC-CoA, succinyl-CoA; SUC, succinate; FUM, fumarate;

MAL, malate; OAA, oxaloacetate; LYS, L-lysine; E4P, erythrose 4-phosphate; DAHP, 3-deoxy-D-arabinoheptulosonate 7-phosphate; DHQ, 3-dehydroquinate; DHS, 3-dehydroshikimate; SHK, shikimate; S3P, shikimate-3-phosphate; EPSP, 5-enolpyruvyl-shikimate 3-phosphate; CHA, chorismate; L-TYR, L-tyrosine; L-PHE, L-phenylalanine; Gln, glutamine; Glu, glutamate; ANT, anthranilate; PRANT, *N*-(5-phosphoribosyl)-anthranilate; L-TRP, L-tryptophan; HCYS, L-homocysteine; MET, L-methionine; SAM, *S*-adenosyl-L-methionine; SAH, *S*-adenosyl-L-homocysteine; SRH, *S*-ribosyl-L-homocysteine; SER, L-serine; ASP, L-aspartate; ILE, isoleucine; MANT, methyl anthranilate; QA, quinate; PCA, protocatechuate. Enzymes that encode the genes listed are provided in Supplementary Table 7. **c**, Knockdown of target genes using the BHR-sRNA system for MANT production. $n = 2$ biologically independent samples. **d**, Effect of changing the start codon of the *GFP* gene on the fluorescence. WT *C. glutamicum* strain harboring pCS-I16-GFP, pCS-I16-GFP (GTG), pCS-I16-GFP (TTG). Abbreviation: NC, WT *C. glutamicum* strain harboring pCS. Error bars, mean ± SD ($n = 3$ biologically independent samples). arb. units: arbitrary units. **e**, Effect of the knockdown of top three target genes (by exchanging their stop codons from ATG to GTG in the chromosome) on the production of MANT. Abbreviation: CT, control strain *C. glutamicum* DBDH/pCS-amGH harboring pEKEx1. Error bars, mean ± SD ($n = 3$ biologically independent samples). $^*P < 0.05$, determined by two-tailed Student's *t*-test. Source data are provided as a Source Data file.

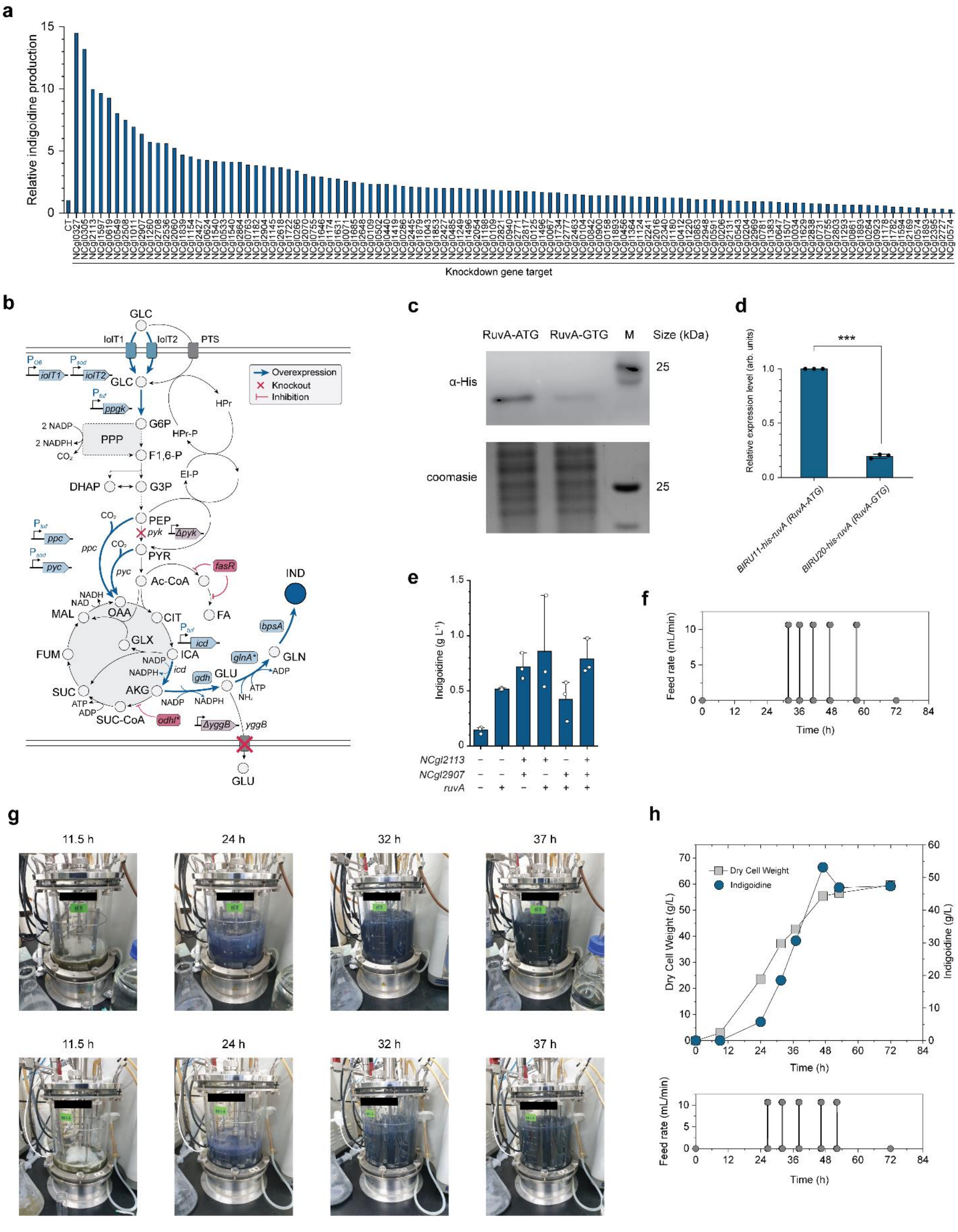
a
Relative indigoidine production
Knockdown gene target
CT
b
GLC
IolT1
IolT2
PTS
Overexpression
Knockout
Inhibition
iolT1
iolT2
ppgk
HPr
G6P
HPr-P
2 NADP
2 NADPH
PPP
F1,6-P
DHAP
G3P
EI-P
PEP
pyk
Δpyk
ppc
pyc
PYR
fasR
Ac-CoA
FA
IND
MAL
OAA
CIT
GLX
ICA
icd
FUM
NADPH
SUC
AKG
GLU
GLN
bpsA
glnA*
gdh
ADP
ATP
SUC-CoA
odhI*
ΔyggB
yggB
c
RuvA-ATG
RuvA-GTG
M
Size (kDa)
α-His
coomasie
25
d
***
Relative expression level (arb. units)
BIRU11-his-ruvA (RuvA-ATG)
BIRU20-his-ruvA (RuvA-GTG)
e
Indigoidine (g L⁻¹)
NCgl2113
NCgl2907
ruvA
f
Feed rate (mL/min)
Time (h)
g
11.5 h
24 h
32 h
37 h
h
Dry Cell Weight
Indigoidine
Dry Cell Weight (g/L)
Indigoidine (g/L)
Time (h)
Feed rate (mL/min)

Supplementary Fig. 7. Colorimetric screening of indigoidine overproducers by employing the genome-scale BHR-sRNA library. **a**, Initial high-throughput screening of indigoidine overproducing *C. glutamicum* strains (108 strains selected from ~68,700 colonies). CT, control strain WT/pCS-bpsA. **b**, Pathways depicting engineering performed on the engineered *C. glutamicum* BIRU11 strain capable of producing indigoidine. **c**, Representative western-blot and SDS-PAGE bands from triplicate experiments on the BIRU11-his-ruvA and BIRU20-his-ruvA strains. Arrow denotes the RuvA protein band. M, protein size marker. **d**, The RuvA levels in the BIRU11-his-ruvA and the BIRU20-his-ruvA strains. Target protein levels were estimated by measuring the intensities of western-blot bands using the Image Lab software. Measured western-blot band intensities were normalized to the total intensity of the Coomassie-stained SDS-PAGE bands. ***P < 0.001, determined by two-tailed Student's *t*-test. arb. units: arbitrary units. **e**, Indigoidine production by *C. glutamicum* WT-BpsA introduced with plasmids harboring two or three sRNAs targeting the genes noted as '+'. **f**, Glucose feed profile during the fed-batch fermentation of the engineered *C. glutamicum* BIRU20 strain for the production of indigoidine in Fig. 3h. **g**, Time lapse photos of the fermenter during the fed-batch fermentation of the BIRU20 strain. The upper photos correspond to the time-lapse photos of fermentation shown in Fig. 3h. The lower photos correspond to the time-lapse photos of fermentation shown in panel h. **h**, A duplicate independently performed fed-batch fermentation of BIRU20 to produce indigoidine from glucose. Symbols: blue circle, indigoidine concentration (g L^{-1}); grey square, dry cell weight (g L^{-1}). (**d,e**) Error bars, mean ± SD (n = 3 biologically independent samples). Source data are provided as a Source Data file.

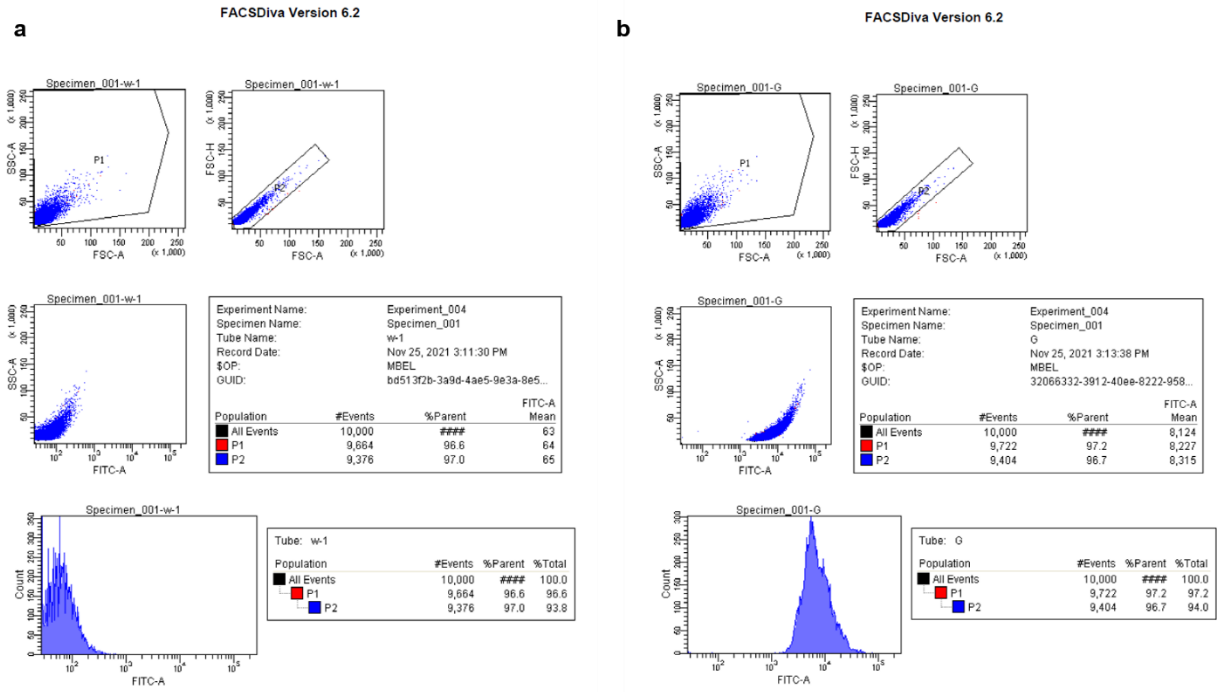

Supplementary Fig. 8. A figure exemplifying the gating strategy for flow cytometry. a, Gating strategy used for wild-type *C. glutamicum* strain. **b**, Gating strategy used for *C. glutamicum* strain expressing *GFP*.

Supplementary Table 1. List of all previous applications of synthetic sRNA in bacteria.

Bacteria	sRNA scaffold	Reference
Escherichia coli	MicC from *E. coli*	[1-5]
Escherichia coli	SgrS-S from *E. coli*	[6]
Shewanella oneidensis	MicC from *E. coli*	[7]
Pseudomonas putida	MicC from *E. coli*	[8]
Bacillus subtilis	MicC from *E. coli*	[9]
Clostridium acetobutylicum	MicC from *E. coli*	[10]
Corynebacterium glutamicum	MicC from *E. coli*	[11]
Synechocystis sp. PCC 6803	MicC from *E. coli*	[12]
Synechococcus elongatus	MicC from *E. coli*	[13]
Halomonas bluephagenesis	PrrF1 from *Pseudomonas aeruginosa*	[14]

Supplementary Table 2. Nucleotide sequences of sRNA scaffolds used in this study.

Scaffold	Sequence	Organism
MicC	tttctgttgggccattgcattgccactgattttccaacatataaaaagacaagcccgaa cagtcgtccgggcttttttt	*E. coli*
SprX2	tcacccaagcatgtcactgggtgtttttcttacgatagagagca	*S. aureus*
RoxS	acatatgaaaccgcgcttatcccggcgcggtttcttt	*B. subtilis*
LhrA	gccgtgtagttcatgtctatgcctcatcccgttatgcataggcgcccgaacatgatgg caacgctggaaatgtttatttcgagcgtcttttttt	*L. monocytogenes*
Scr5239	gagagggctgagggtccctctcaggcgc	*S. coelicolor*
BtsR1	tttacatgtattgtaaaccttcacactaatgtgaagg	*B. thuringiensis*
ArnA	aaaaaattacccctgaatagcctttaaaagactgttcgggggtaacgatgt	*C. glutamicum*

Supplementary Table 3. Sequences of the Hfq proteins used in this study.

Hfq source	Sequence
E. coli	MAKGQSLQDPFLNALRRERVPVSIYLVNGIKLQGQIESFDQFVILLKNTV SQMVYKHAISTVVPSRPVSHHSNNAGGGTSSNYHHGSSAQNTSAQQDS EETE
S. aureus	MIANENIQDKALENFKANQTEVTVFFLNGFQMKGVIEEYDKYVVSLNSQ GKQHLIYKHAISTYTVETEGQESTESEE
L. monocytogenes	MKQGGQGLQDYYLNQLRKEKILATVFLTNGFQLRGRVVSFDNFTVLLD VEGKQQLVFKHAISTFSPQKNVALNPDAE
B. thuringiensis	MEHLQEELYKQIKEEKGTVTIFLKSGVRIVGEVVGVDKFTVLILVDGKQ QLIYKQAISTIMK
B. subtilis	MKPINIQDQFLNQIRKENTYVTVFLLNGFQLRGQVKGFDNFTVLLESEG KQQLIYKHAISTFAPQKNVQLELE

Supplementary Table 4. Comparison of the BHR-sRNA system with CRISPRi and the MicC-EcHfq system.

	**CRISPRi**	**MicC-EcHfq**	**RoxS-BsHfq (BHR-sRNA)**
Mechanism of action	Transcriptional repression	Translational repression	Translational repression
Size of the protein[a]	Cas9 or Cas12a (1000–1500 aa)<br>Cas12f (400–700 aa)<br>Cas12j (700–800 aa)	EcHfq (102 aa)[b]	BsHfq (73 aa)[b]
Applicability to broad-host bacteria	Yes	Yes (preferentially Gram-negative bacteria)	Yes
Applicability to eukaryotes	Yes	No	No
Target binding sequence design	Consideration of PAM sequences required	24 nt from the start codon	24 nt from the start codon

[a] aa, amino acid.

[b] Corresponds to the monomer size; Hfq proteins form hexamers inside the cell.

Supplementary Table 5. Reporter genes and plasmids employed for testing the BHR-sRNA system in diverse bacteria.

Organism	Reporter	Plasmids
L. lactis	*upp* in genome<br>(susceptibility to 5-fluorouracil)	pTD6-RoxS-anti-upp
S. epidermidis	mRFPmars on plasmid	pSK9065 (reporter)<br>pRMCbhr-anti-mRFPmars
B. subtilis	*EGFP* in genome	pBsuCGroxS-antiEGFP
S. coelicolor	*act* operon in genome<br>(actinorhodin biosynthesis)	pGMbhr-anti-actIORFI
R. opacus	*EGFP* on plasmid	pCESAct-EGFP (reporter)<br>pEK-bhr-antiEGFP
C. xerosis	*GFP* on plasmid	pCS-I16-GFP (reporter)<br>pEK-bhr-antiGFP
C. glutamicum ATCC 13032	*GFP* on plasmid	pCS-I16-GFP (reporter)<br>pEK-bhr-antiGFP
C. glutamicum BE	*GFP* on plasmid	pCS-I16-GFP (reporter)<br>pEK-bhr-antiGFP
C. necator	*EGFP* on plasmid	pACYC-184-EGFP (reporter)<br>pEK-bhr-antiEGFP
C. violaceum	*vio* operon in genome<br>(violacein biosynthesis)	pBBR1bhr-anti-vioA
P. putida	*EGFP* in genome	pBBR1bhr-anti-EGFP
V. natriegens	*EGFP* on plasmid	pACYC-EGFP (reporter)<br>pEK-bhr-antiEGFP
A. hydrophilia	*GFP* on plasmid	pCS-tuf-GFP (reporter)<br>pEK-bhr-antiGFP
K. pneumoniae	*EGFP* on plasmid	pACYC-184-EGFP (reporter)<br>pEK-bhr-antiEGFP
E. coli Nissle 1917	*EGFP* on plasmid	pACYC-EGFP (reporter)<br>pEK-bhr-antiEGFP
E. coli DH5α	*EGFP* on plasmid	pACYC-EGFP (reporter)<br>pEK-bhr-antiEGFP

Supplementary Table 6. Gene knockdown targets tested for enhanced valerolactam production in *C. glutamicum*.

No.	Gene ID	Gene name	Encoded enzyme	First 24 nt
1	NCgl1136	*hom*	Homoserine dehydrogenase	atgacctcagcatctgccccaagc
2	NCgl0634	*icd*	Isocitrate dehydrogenase	atggctaagatcatctggacccgc
3	NCgl1999	*gdh*	NADP-specific glutamate dehydrogenase	atgacagttgatgagcaggtctct
4	NCgl0950	*aroF*	Probable phospho-2-dehydro-3-deoxyheptonate aldolase	atgagttctccagtctcactcgaa
5	NCgl2098	*aroG*	Phospho-2-dehydro-3-deoxyheptonate aldolase	atgaatagggggtgtgagttggaca
6	NCgl2167	*aceE*	Pyruvate dehydrogenase E1 component	atggccgatcaagcaaaacttggt
7	NCgl2521	*pqo* (*poxB*)	Pyruvate dehydrogenase	atggcacacagctacgcagaacaa
8	NCgl2008	*pyk1*	Pyruvate kinase	atgggcgtggatagacgaactaag
9	NCgl2083	*murE*	Probable UDP-N-acetylmuramoylalanyl-D-glutamate—2,6-diaminopimelate ligase	atggcaaccacgttgctggacctc
10	NCgl0233	*gltX* (*crp*)	Glutamyl-tRNA synthetase-related protein	atggcctgcatgactgatgttcgt
11	NCgl0795	*gltA*	Citrate synthase	atgtttgaaagggatatcgtggct
12	NCgl2200	*zur* (*fur*)	Ferric uptake regulation protein	gtgggtatcaatcgcatcagccaa

Supplementary Table 7. Gene knockdown targets tested for enhanced MANT production in *C. glutamicum.*

No.	Target selection method	Gene ID	Gene name	Encoded enzyme	First 24 nt
1	*in-silico*	NCgl1513	*tal*	Transaldolase	atgtctcacattgatgatcttgca
2	*in-silico*	NCgl1396	*gnd*	6-Phosphogluconate dehydrogenase	atgactaatggagataatctcgca
3	*in-silico*	NCgl0414	*hemD*	Uroporphyrinogen III synthase/methyltransferase	atgactatcgcccataagcccgag
4	*in-silico*	NCgl1868	*dapF*	Diaminopimelate epimerase	gtgaatttgaccatcccctttgcc
5	*in-silico*	NCgl1516	*pgl*	Putative 6-phosphogluconolactonase	atggttgatgtagtacgcgcacgc
6	*in-silico*	NCgl0819	*csm*	Putative chorismate mutase	atgactaatgcaggtgacaacttc
7	*in-silico*	NCgl1512	*tkt*	Transketolase	ttgaccaccttgacgctgtcacct
8	*in-silico*	NCgl0403	*hemC*	Porphobilinogen deaminase	atgaccttaaaaattggtacccga
9	*in-silico*	NCgl1379	-	Predicted divalent heavy-metal cations transporter	atggatacctcgactgtgttgttc
10	*in-silico*	NCgl1523	*ppc*	Phosphoenolpyruvate carboxylase	atgactgatttttacgcgatgac
11	Rational	NCgl0286	*crp*	Transcriptional regulator, Crp/Fnr family	gtggaaggtgtacaggagatcctg
12	Rational	NCgl2799	*pheA*	Prephenate dehydratase	atgagcgacgcaccaactgttgtg
13	Rational	NCgl0223	*tyrA*	Putative prephenate dehydrogenase	gtgactaccaaagacatttcccgc
14	Rational	NCgl0795	*gltA*	Citrate synthase	atgtttgaaagggatatcgtggct
15	Rational	NCgl2200	*zur*	Ferric uptake regulation protein	gtgggtatcaatcgcatcagccaa

Supplementary Table 8. Top 13 genes screened to increase indigoidine production in *C. glutamicum*.

No.	Gene ID	Gene name	Encoded enzyme	Fold-increase	First 24 nt
1	*NCgl 0327*	*rmlB1*	dTDP-Glucose 4,6-dehydratase	14.5	atgacttctttgcttgtgaccgga
2	*NCgl 0305*	-	Hypothetical protein	13.2	atgctgaacgggttcgtgaccaaa
3	*NCgl 2113*	-	Hypothetical protein	9.9	gtgggggagtgggcggacatgaat
4	*NCgl 1597*	*ruvA*	Holliday junction resolvasome DNA-binding subunit	9.6	atgattgcctcacttcgtggcact
5	*NCgl 0619*	*spoU*	Putative tRNA/rRNA methyltransferase protein	9.3	atgccggaacacccacttcacgtt
6	*NCgl 0549*	-	Membrane protein	8.0	atgggcacgatgcgagaaatggtc
7	*NCgl 2508*	*purC*	Phosphoribosylaminoimidazole-succinocarboxamide synthase	7.5	atgcgtcctgaactctcccagtac
8	*NCgl 1011*	-	Hypothetical protein	6.9	atggccacttcacattcacgaccc
9	*NCgl 2907*	-	Putative membrane protein	6.4	atgtcaaccattcacgcctccgga
10	*NCgl 1260*	-	Putative metal dependent phosphohydrolase, *relA/spoT* homolog	5.7	gtgaatacactttccccgcgcctt
11	*NCgl 2708*	-	Hypothetical protein	5.6	atgaccgttgtatcgcacgcgctt
12	*NCgl 2536*	-	Putative membrane protein	5.6	atgaaccgcgcacgaatcgcgacc
13	*NCg l2060*	-	ATPase component of ABC transporters with duplicated ATPase domains	5.2	atgcgcacttttgccgcttatatt

Supplementary Table 9. Comparison of growth rate and indigoidine production during the fed-batch cultures of BIRU11 and BIRU20 strains.

Strain	BIRU11[a]	BIRU20[b]
Titer (g L^{-1})	49.3	54.9
Productivity (g L^{-1} h^{-1})	0.967	1.04
Content (g g^{-1})[c]	0.758	0.840
Yield (mol mol^{-1})[d]	0.1	0.181
Maximum specific growth rate (g L^{-1} h^{-1})	3.70	1.91
Maximum specific productivity (g L^{-1} h^{-1})	2.30	2.77

[a] Data from Fig. 4a of Ghiffary *et al.*[15]
[b] Data from Fig. 3h
[c] g(indigoidine) $gDCW^{-1}$
[d] mol(indigoidine) mol(consumed $glucose)^{-1}$

Supplementary Method 1. Construction of plasmids used in this study

The sRNA cassettes were constructed based on the pEKEx1 plasmid. First, *E. coli*-derived sRNA platforms were tested. *E. coli hfq* (*Echfq*) with or without codon optimization for expression in *C. glutamicum* were inserted into pEKEx1. pEK-Echfq was constructed by PCR amplification of the *Echfq* gene from the genomic DNA of *E. coli* W3110 using primers Echfq_gib_F and Echfq_gib_R and inserting into pEKEx1 at EcoRI site by Gibson assembly[16]. pEK-Echfq(opt) was constructed similarly by PCR amplification of the codon optimized hfq gene from the artificially synthesized *Echfq(opt)* gene using primers Echfq_opt_gib_F and Echfq_opt_gib_R and inserting into pEKEx1 at EcoRI sites by Gibson assembly. To construct different sRNA constructs without target binding sequence, sRNA scaffold MicC was PCR amplified from the pWAS plasmid using primers M9A1_MicC_F (with a 5'-extension containing *tac* promoter sequence) and M5A_R (with a 5'-extension containing sequences suitable for Gibson assembly), which was again PCR amplified using primers M9K_F and M5E_R. The amplified gene fragment was inserted into pEKEx1, pEK-Echfq, and pEK-Echfq(opt) at StuI site to construct pEK-MicC, pEKehm, and pEK-MicC-Echfq(opt), respectively. Construction of sRNA plasmids harboring the anti-*GFP* sequence (reverse complementary to the first 24 nt of *GFP*) was performed similarly. The MicC-based sRNA fragment harboring the anti-*GFP* sequence was PCR amplified using primers M9A1_antiGFP_F and M5A_R, which was again PCR amplified using primers M9K_F and M5A_R. The amplified gene fragment was inserted into pEKEx1, pEK-Echfq, and pEK-Echfq(opt) at StuI site to construct pEK-MicC-antiGFP, pEK-ehm-antiGFP, and pEK-MicC-Echfq(opt)-antiGFP, respectively.

To construct pCS and pCS-I16-GFP (the plasmid containing the reporter *GFP* gene), the antibiotic markers (Km^R) of the plasmids pCES208 and pCES-I16-GFP were exchanged with Spc^R. This was done by linearizing the plasmids pCES208 and pCES-I16-GFP by inverse PCR using primers 208_inv_F and 208_inv_R, and inserting Spc^R (amplified from pTacCC1 using primers spc_gib_F and spc_gib_R) by Gibson assembly. This completed the construction of pCS and pCS-I16-GFP, respectively. The construction *mCherry* reporter plasmid was done in the following manner. Gene *mCherry* was amplified using primers pCES-mCherry-F and pCES-mCherry-R and cloned to XbaI and BamHI double-digested plasmid pCS to construct pCES-mCherry.

To construct plasmids containing sRNA constructs from different Gram-positive bacteria, pWAS (the plasmid containing MicC-based sRNA construct) derivatives containing different sRNA scaffolds were firstly cloned. pWAS harboring *C. glutamicum* ArnA-T1/TE terminator (pWAS-ArnA) was constructed by linearizing pWAS by inverse PCR using primers pWAS_ArnA_IV_F and pWAS_ArnA_IV_R (each containing a portion of the ArnA scaffold sequence), followed by DpnI treatment and consequent ligation of the linearized vector with T4 PNK and T4 ligase. pWAS-SprX2, pWAS-RoxS, pWAS-LhrA, pWAS-Scr5239, and pWAS-BtsR1 were constructed similarly using primer pairs pWAS_SprX2_IV_F/pWAS_SprX2_IV_R, pWAS_RoxS_IV_F/pWAS_RoxS_IV_R, pWAS_LhrA_IV_F/pWAS_LhrA_IV_R, pWAS_Scr5239_IV_F/pWAS_Scr5239_IV_R, and pWAS_BtsR1_IV_F/pWAS_Scr5239_IV_R. Before construction of pEKEx1-based sRNA constructs, different *hfq* genes were firstly inserted. pEK-Sahfq was constructed by PCR amplification of the artificially synthesized *Sahfq* from *S. aureus* using primers Sahfq_gib_F and Sahfq_gib_R, and was inserted at EcoRI and PstI sites of pEKEx1. pEK-Bshfq was constructed by PCR amplification of the *Bshfq* gene from the genomic DNA of *B. subtilis* using primers Bshfq_gib_F and Bshfq_gib_R, and was inserted at EcoRI and PstI sites of pEKEx1 to construct pEK-Sahfq. pEK-LmHfq and pEK-BtHfq were constructed by inserting the artificially synthesized *LmHfq* from *L. monocytogenes* and *BtHfq* from *B. thuringiensis* at EcoRI and PstI sites of pEKEx1, respectively. Construction of sRNA plasmids harboring the anti-*GFP* sequence (reverse complementary to the first 24 nt of *GFP*) was performed as follows. The ArnA, SprX2, RoxS, LhrA, Scr5239, and BtsR1-based sRNA fragments harboring the anti-*GFP* sequence were PCR amplified using pWAS-ArnA, pWAS-SprX2, pWAS-RoxS, pWAS-LhrA, pWAS-Sco5239, and pWAS-BtsR1 as the template, respectively, using primer sets M9A1_ArnA_GFP_F/M5A_R, M9A1_SprX2_GFP_F/M5A_R, M9A1_RoxS_GFP_F/M5A_R, M9A1_LhrA_GFP_F/M5A_R, M9A1_Sco_GFP_F/M5A_R, and M9A1_BtsR1_GFP_F/M5A_R, respectively, which were again PCR amplified using primers M9K_F and M5A_R. To construct pEK-ArnA-antiGFP and pEK-Scr5239-antiGFP, the ArnA-antiGFP fragment and the Scr5239-antiGFP fragment were inserted into pEKEx1 at StuI site, respectively. To construct pEK-shs-antiGFP and pEK-SprX2-antiGFP, the SprX2-antiGFP fragment was inserted into pEK-SaHfq and pEKEx1 at StuI site, respectively. To construct pEK-bhr-antiGFP and pEK-RoxS-antiGFP, the RoxS-antiGFP fragment was inserted into pEK-BsHfq and pEKEx1 at StuI site, respectively. To

construct pEK-lhl-antiGFP and pEK-LhrA-antiGFP, the LhrA-antiGFP fragment was inserted into pEK-LmHfq and pEKEx1 at StuI site, respectively. To construct pEK-bhb-antiGFP and pEK-BtsR1-antiGFP, the BtsR1-antiGFP fragment was inserted into pEK-BtHfq and pEKEx1 at StuI site, respectively.

To construct pEK-bhr derivatives harboring anti-*sfGFP* constructs with varying lengths of anti-*sfGFP* target binding sequences (from 16 nt to 40 nt), similar procedures were performed as described above. First, anti-*sfGFP* sRNA constructs were amplified from pWAS-RoxS using primers M9A1_RoxS_sfGFP_NN_F (NN corresponds to the length of the target binding sequence) and M5A_R, which was again PCR amplified using primers M9K_F and M5E_R. The amplified gene fragments were inserted to pEK-Bshfq at StuI site using Gibson assembly to construct pEK-bhr-antiSfGFP-NNnt.

Construction of CRISPRi plasmids were performed in the following manner[17]. Briefly, the anti-GFP sgRNA construct was amplified from pUC-sgRNA using primers M9-GFP20_1F and M5E_R, which was again PCR amplified using primers M9K_F and M5E_R. The resulting amplified fragment was inserted to pEK-dCas9-opt at StuI site using Gibson assembly to construct pEK-dCas9-GFP. Construction of CRISPRi-BHR-sRNA dual targeting plasmid was performed in the following manner. The gene *hfq* from *B. subtilis* was amplified from plasmid pEK-Bshfq using primers pEK-BsHfq-avrII-F and pEK-BsHfq-avrII-R and cloned to pEK-dCas9-GFP at the AvrII site to construct pEKEx1-dCas9-sgRNA(GFP)-BsHfq. The sRNA construct containing anti-GFP was amplified from pEK-bhr-anti-GFP using primers pEK-antiGFP-sacII-F and antiGFP-sacII-R and cloned to plasmid pEKEx1-dCas9-sgRNA(GFP)-BsHfq at the SacII site to construct pEKEx1-dCas9-sgRNA(GFP)-BsHfq-antiGFP-sRNA.

Construction of GFP plasmids with start codon changed to GTG and TTG were performed in the following manner. First, the GFP (A1G) and GFP (A1T) genes were amplified from pCS-I16-GFP using primer pairs I16_GFP_G_F/I16_GFP_R and I16_GFP_T_F/I16_GFP_R. The gene fragments were cloned to the pCS-I16 backbone (linearized with ivp_16_F and ivp_16_R) using Gibson assembly to construct pCS-I16-GFP (GTG) and pCS-I16-GFP-(TTG).

Construction of sRNA-containing plasmids for knockdown of fluorescence genes were performed in a similar manner described above. The sRNA construct containing anti-*EGFP* was amplified from pWAS-RoxS using primers M9A1_RoxS_EGFP_F and M5A_R, which was again PCR amplified using primers M9K_F and M5E_R. The amplified gene fragment was inserted to pEKEx1, pEK-Bshfq, and pEK-Echfq at StuI site to construct pEK-RoxS-antiEGFP, pEK-bhr-antiEGFP, and pEK-ehm-antiEGFP, respectively. Construction of *mCherry* and *mCherry-sfGFP* double targeting sRNA plasmids were performed similarly. The sRNA construct containing anti-*mCherry* was amplified from pWAS-RoxS using primers M9A1_roxS_mCherry and M5E_R, which was again PCR amplified using primers M9K_F and M5E_R. The amplified gene fragment was inserted to pEK-Bshfq at StuI site to construct pEK-bhr-anti-mCherry. The sRNA fragment *Bs*Hfq-anti-*mCherry* was PCR amplified using primers CglsRNA_frag_AvrII_F and CGlsRNA_frag_AvrII_R and the resulting PCR product was cloned to pEK-bhr-anti-*sfGFP* at the AvrII site to construct pEK-bhr-anti-*mCherry*-anti-*sfGFP*.

To test the performance of the sRNA platform in *B. subtilis*, plasmids pBsuCGroxS-antiEGFP was constructed. To construct the vectors, the *cat*-pBR322-*bla* fragment of plasmid pB0A[18] was amplified using primers pB0ArepUTR- and pBusC-. In addition, the *rep* gene of plasmid pB0A[18] of which TTG start codon was substituted with ATG start codon [rep(ATG) fragment] was amplified using primers pB0A-ATG-rep+ and pB0A-rep-. Subsequently, the *cat*-pBR322-*bla* and the rep(ATG) fragments were used as templates to amplify a single DNA fragment pBus-rep(ATG) using primers pB0A-rep- and pBsuC-. To prepare sRNA expression cassette, antiEGFP-roxS fragment was amplified from pEK-bhr using primer pair antiEGFP-roxS-SL3+/rep(Bsu)-T1TE-. In addition, PgroES fragment was amplified from the genomic DNA of *B. subtilis* 168 using primer pairs pBsuC-PgroES+/antiEGFP-PgroES-. Next, PgroES-roxS fragment was amplified with primers pBsuC-PgroES+ and rep(Bsu)-T1TE- using DNA fragments PgroES and antiEGFP-roxS as templates. Finally, the DNA fragment PgroES-roxS was respectively assembled with the DNA fragment pBsu-rep(ATG) through Gibson assembly, resulting in pBsuCGroxS-antiEGFP.

To construct a reporter plasmid for expression in *R. opacus*, pCHAct was firstly constructed for construction of gene expression cassettes. Three gene fragments are prepared: (1)

the plasmid pCH (ref. [19]) was digested with BamHI and BglII; (2) *amiCADS* operon as an acetamide-inducible promoter cassette was PCR amplified from the genomic DNA of *Mycobacterium smegmatis* using primers amiCADS_F and amiCADS_R; (3) *rrnB* terminator was PCR amplified from pTac15K using primers rrnB_F and rrnB_R. These three gene fragments were assembled to construct pCHAct using Gibson assembly. Then, pCESAct was constructed based on pCS for expression in a Spc-resistant plasmid. The acetamide-inducible promoter-multiple cloning site-*rrnB* terminator fragment was PCR amplified from pCHAct using primers pAct_F and pAct_R which was assembled with pCS linearized by inverse PCR using primers pCES_IV_F and pCES_IV_R. Then, to construct pCESAct-EGFP, *EGFP* was PCR amplified from pACYC-EGFP using primers pCESAct-EGFP_F and pCESAct-EGFP_R and was inserted into pCESAct at SpeI and NdeI sites by Gibson assembly. To construct pCESAct-rppA, the *rppA* gene was amplified from pTac-5'UTR-Sgr_rppA using primers pCESAct-rppA_F and pCESAct-rppA_R and was inserted into pCESAct at SpeI and NdeI sites by Gibson assembly.

To test the performance of the sRNA platform in *S. coelicolor*, pGM-BsHfq harboring *B. subtilis hfq* was first constructed by employing the previously constructed pCRISPR-Cas9 plasmid[20] as the backbone plasmid. pCRISPR-Cas9 was linearized by PCR amplification using primers pCRISPR_IV_F and pCRISPR_IV_R and was assembled (by Gibson assembly) with the *BsHfq* fragment PCR amplified from pEK-BsHfq using primers BsHfq_Sco_F and BsHfq_Sco_R. Then, RoxS-based sRNA constructs were amplified from pWAS-RoxS using primer pair Sco_RoxS_actIORFI/Sco_sRNA_R. The amplified RoxS-based sRNA fragment was inserted to pGM-BsHfq at NcoI site using Gibson assembly to construct pGMbhr-anti-actIORFI. As a control plasmid, pGM-CTRL plasmid was also constructed without *hfq* nor sRNA; the previously obtained linearized pCRISPR-Cas9 blunt-end ligated using T4 PNK and ligase.

To test the performance of the sRNA platform in *S. epidermidis*, pRMC2-BsHfq harboring *B. subtilis hfq* was first constructed by employing the pRMC2 plasmid[21] as the backbone plasmid. During cloning, *E. coli* cells harboring pRMC2 derivatives were selected in LB agar plates supplemented with 100 μg mL^{-1} of Ap while *S. epidermidis* strains harboring the corresponding plasmids were selected using 17 μg mL^{-1} of Cm. The BsHfq fragment amplified from pEK-BsHfq using primers pRMC2_BsHfq_F and pRMC2_Hfq_R was inserted to pRMC2 at KpnI site by

Gibson assembly to construct pRMC2-BsHfq. Then, RoxS-based sRNA constructs targeting *mRFPmars*, *icaA*, and *sarA* were amplified from pWAS-RoxS using primer pairs RMCRoxS_antiRFP_F/pRMC2_sRNA_R1, RMCRoxS_icaA_F/pRMC2_sRNA_R1, and RMCRoxS_sarA_F/pRMC2_sRNA_R1, which were PCR amplified using primers pRMC2_sRNA_F2 and pRMC2_sRNA_R2. The amplified RoxS-based sRNA fragments were inserted to pRMC2-BsHfq at SphI site using Gibson assembly to construct pRMCbhr-anti-mRFPmars, pRMCbhr-anti-icaA, and pRMCbhr-anti-sarA.

To test the performance of the sRNA platform in *L. lactis*, pTD6-RoxS-anti-upp harboring *B. subtilis* sRNA *RoxS* were constructed to target the *upp* gene on the *L. lactis* genome. The RoxS-based anti-*upp* sRNA construct was amplified from pEK-bhr. The primer pair LAB-sRNA_bs_LL_F/LAB-sRNA_R was used to amplify RoxS-based sRNA construct targeting *upp*. Extension PCR was done using primer pairs LAB-hfq_ext_F/LAB-sRNA_R and cloned to backbone plasmid pTD6 (linearized using primer pairs LAB_ivp_pTD6_F/LAB_ivp_pTD6_R) using Gibson assembly to construct pTD6-RoxS-anti-upp.

To test the performance of the sRNA platform in *E. coli*, *K. pneumoniae,* and *C. necator*, plasmid pACYC-EGFP was constructed. The *EGFP* gene fragment was amplified from pES0-EGFP using primers EGFP_PacI_F and EGFP_XhoI_R, and was inserted into pACDsRed2 at PacI and XhoI sites by restriction enzyme digestion and followed by ligation. The sRNA plasmids pEK-ehm-antiEGFP and pEK-bhr-antiEGFP were used for the abovementioned strains.

To test the performance of the sRNA platform in *P. putida*, *C. violaceum,* and *R. sphaeroides*, pBBR1TaKL-BsHfq harboring *Bshfq* was first constructed by employing the Gram-negative broad-host-range pBBR1MCS-2 (ref. [22]) plasmid as the backbone plasmid. To construct pBBR1TaKL-BsHfq, the *lacI-BsHfq* fragment amplified from pEK-BsHfq using primers pTacLac_pBBR1_F and pTacLac_pBBR1_R was inserted to pBBR1MCS-2 which was linearized by inverse PCR using primers pBBR1TaKL_IV_F and pBBR1TaKL_IV_R. Then, RoxS-based anti-*EGFP* and anti-*vioA* sRNA constructs were amplified from pWAS-RoxS using primer pairs RoxS_EGFP_F/sRNA_AgeI_R and RoxS_vioA_F/sRNA_AgeI_R, which were again PCR amplified using primers sRNA_AgeI_F2 and sRNA_AgeI_R2. The amplified RoxS-based sRNA

fragments were inserted to pBBR1TaKL-BsHfq at AgeI site using Gibson assembly to construct pBBR1bhr-anti-EGFP and pBBR1bhr-anti-vioA, respectively.

To construct pEK-bhr derivatives harboring sRNAs containing target binding sequences, similar procedures were performed as described above. First, anti-GOI (GOI stands for gene of interest) sRNA constructs were amplified from pWAS-RoxS using primers M9A1_roxS_GOI_F and M5A_R, which was again PCR amplified using primers M9K_F and M5E_R. The amplified gene fragments were inserted to pEK-Bshfq at StuI site using Gibson assembly to construct pEK-bhr-anti-GOI.

To construct pCS-ABact, primers hisact_Not_F and hisact_Not_R were used to amplify the *act* gene from pET−30α(+)-his-act, and the resulting amplified fragment was cloned to pGA1 digested with NotI.

To construct pCS-amGH, primers g_amGH_F and g_amGH_R were used to amplify the *aamt1*$^{opt\text{-}Cgl}$ fragment from pSH36HTc, and the amplified fragment was cloned to at the NotI site of pEKGH.

To construct pCS-bpsA, the backbone plasmid pCS was first linearized and amplified using primers grna2_F and grna2_R. Primers bpsa_F1 and bpsa_R2 were used to amplify the bpsA gene from pEK-BA1.

To construct plasmids harboring two sRNAs, a receiver sRNA plasmid was first digested with AvrII, followed by insertion by Gibson assembly of another sRNA fragment amplified from a donor sRNA plasmid (using primers sRNA_frag_AvrII_F and sRNA_frag_AvrII_R). To construct plasmids harboring three sRNAs, a receiver sRNA plasmid constructed above (harboring two sRNAs) digested with AvrII was inserted by Gibson assembly with an sRNA fragment amplified from a donor sRNA plasmid (using primers sRNA_Triple_AvrII_F and sRNA_frag_AvrII_R).

Supplementary Method 2. Knockdown of reporter genes in diverse bacteria

Knockdown of reporter genes in *Bacillus subtilis*

For two-step replacement of a chromosomal region spanning from the *sigF* gene to the *spoVAF* gene with *EGFP* or no gene in *B. subtilis*, linear DNA fragments were prepared to modify the chromosome of *B. subtilis* through natural competence. To prepare the first DNA fragment ΔsigF-lysA::cat for replacing the *sigF-lysA* region with the *cat* gene responsible for chloramphenicol resistance, a pair of 1-kb homology arms sigFup and lysA3' were amplified from the genomic DNA of *B. subtilis* 168 using primer pairs del_sigF_L+/del_sigF_L- and del_sigF_R+/del_sigF_R-, respectively. In addition, the del_sigF-cat fragment was amplified from plasmid pB0A using primers del_sigF-catBsu+ and del_sigF-catBsu-. Subsequently, the DNA fragment ΔsigF-lysA::cat was amplified using primers del_sigF_L+ and del_sigF_R- using the three DNA fragments sigFup, lysA3', and del_sigF-cat as templates.

To prepare the second DNA fragment ΔsigF-spoVAF::EGFP-lysA for replacing the temporarily introduced *cat* gene with the P*grac-egfp-lysA* cassette, the sigFup and lysA fragments were amplified from the genomic DNA of *B. subtilis* 168 using primer pairs del_sigF_L+/del_sigF_L- and spoVAF3+/del_sigF_R-, respectively. In addition, DNA fragments P*grac* and *EGFP* were amplified from plasmids pHCas and pES0-EGFP using primer pairs sigF5-Pgrac+/Pgrac- and Pgrac-EGFP+/lysA-EGFP-, respectively. Subsequently, the DNA fragment P*grac*-EGFP was amplified with primers sigF5-Pgrac+ and lysA-EGFP- using P*grac* and EGFP as templates, and the DNA fragment ΔsigF-spoVAF::EGFP-lysA was amplified with primers del_sigF_L+ and del_sigF_R- using the DNA fragments sigFup, lysA, and Pgrac-EGFP as templates.

To prepare the third DNA fragment ΔsigF-spoVAF::lysA for replacing the temporarily introduced *cat* gene with the *lysA* gene, the sigFup and lysA fragments were amplified from the genomic DNA of B. subtilis 168 using primer pairs del_sigF_L+/del_sigF_L- and del_sigF-spoVAF3+/del_sigF_R-, respectively. Subsequently, the DNA fragment ΔsigF-spoVAF::lysA was amplified with primers del_sigF_L+ and del_sigF_R- using the two DNA fragments as templates.

To analyze GFP fluorescence from *B. subtilis* (Fig. 2b), the strains were inoculated to 5 mL LB media contained in 50 mL conical tubes supplemented with appropriate antibiotics and incubated overnight at 37°C with rotary shaking at 200 rpm. Then, 500 μL of the culture was transferred to fresh 10 mL LB media contained in 50 mL conical tubes supplemented with appropriate antibiotics, and was incubated for 2 h at 37°C with rotary shaking at 200 rpm.

Knockdown of reporter genes in *Rhodococcus opacus*

To analyze GFP fluorescence from *R. opacus* strains (Fig. 2b), the strains were inoculated to 5 mL LB media containing 10 g L^{-1} of glucose contained in 50 mL conical tubes supplemented with appropriate antibiotics and incubated at 30°C with rotary shaking at 200 rpm until the OD_{600} of the cells reached ~4. Then, 200 μL of the culture was transferred to fresh 10 mL LB media containing 10 g L^{-1} of glucose contained in 50 mL conical tubes supplemented with appropriate antibiotics and incubated at 30°C until the OD_{600} of the cells reached ~1. Then, 1 mM of IPTG and 0.17 M of acetamide were added to induce expression of *BsHfq* and *rppA*, respectively. The cells were additionally grown for 48 h. The cells were harvested by centrifugation at 4,000×*g*, washed with PBS, and resuspended to PBS. Subsequently, the resulting samples were transferred to a black, clear-bottom 96-well plate, and OD_{600} and fluorescence intensities were measured using Spark Multimode Microplate Reader.

Knockdown of reporter genes in *C. xerosis* and *C. glutamicum* BE

C. xerosis and *C. glutamicum* BE cells were made electrocompetent with the same protocol as that of *C. glutamicum* ATCC 13032. The electrocompetent *C. xerosis* and *C. glutamicum* BE cells were first transformed with plasmid pCS-I16-GFP via electroporation and plated to BHIS agar plate containing Spc (200 μg mL^{-1}). The transformants were made competent again with the same protocol as above and transformed with plasmids pEKEx1 and pEK-bhr-antiGFP.

To analyze GFP fluorescence from *C. xerosis* and *C. glutamicum* BE (Fig. 2b), the strains were inoculated to 2 mL BHIS media in 14 mL test tubes supplemented with appropriate antibiotics and incubated at 30°C, with rotary shaking at 200 rpm overnight. Then, 20 μL of the

culture was transferred to fresh 2 mL BHIS media supplemented with appropriate antibiotics and 1 mM of IPTG to induce the expression of the sRNA system. The cells were additionally grown for 24 h. The cells were harvested by centrifugation at 4,000×*g*, washed with PBS, and resuspended to PBS. Subsequently, the resulting samples were transferred to a black, clear-bottom 96-well plate, and OD_{600} and fluorescence intensities were measured using Spark Multimode Microplate Reader.

Knockdown of reporter genes in *Klebsiella pneumoniae*

Transformation of *K. pneumoniae* with plasmids was performed by electroporation following the basic protocols as reported[23]. The electrocompetent *K. pneumoniae* cells were first transformed with plasmid pACYC-184-EGFP via electroporation and plated to LB agar plate containing Cm (17 µg mL^{-1}). The transformants were made competent again with the same protocol as above and transformed with plasmids pEKEx1 and pEK-bhr-antiEGFP.

To analyze EGFP fluorescence from *K. pneumoniae* (Fig. 2b), the strains were inoculated to 2 mL LB media in 14 mL test tubes supplemented with appropriate antibiotics and 1 mM of IPTG to induce the expression of the sRNA system, then incubated at 30 °C, with rotary shaking at 200 rpm overnight. The cells were grown for 48 h. The cells were harvested by centrifugation at 4,000×*g*, washed with PBS, and resuspended to PBS. Subsequently, the resulting samples were transferred to a black, clear-bottom 96-well plate, and OD_{600} and fluorescence intensities were measured using Spark Multimode Microplate Reader.

Knockdown of reporter genes in *Aeromonas hydrophila*

Transformation of *A. hydrophila* with plasmids was performed through conjugation[24]. The recombinant plasmid pCS-ptuf-GFP was first introduced into *E. coli* S17-1 by electroporation. *A. hydrophila* was transformed using conjugation with *E. coli* S17-1 harboring pCS-ptuf-GFP, and plated to LB agar plate containing Spc (200 µg mL^{-1}). The transformants were transformed again with the same protocol as above with plasmids pEKEx1 and pEK-bhr-antiGFP.

To analyze GFP fluorescence from *A. hydrophila* (Fig. 2b), the strains were inoculated to 2 mL LB media in 14 mL test tubes supplemented with appropriate antibiotics and 1 mM of IPTG to induce the expression of the sRNA system, then incubated at 30°C, with rotary shaking at 200

rpm overnight. The cells were grown for 48 h. The cells were harvested by centrifugation at 4,000×g, washed with PBS, and resuspended to PBS. Subsequently, the resulting samples were transferred to a black, clear-bottom 96-well plate, and OD_{600} and fluorescence intensities were measured using Spark Multimode Microplate Reader.

Knockdown of reporter genes in *Cupriavidus necator*

Transformation of *C. necator* with vectors was performed by conjugation[24]. The recombinant plasmid pACYC-184-EGFP was first introduced into *E. coli* S17-1 by electroporation. *C. necator* cells were transformed using conjugation with *E. coli* S17-1 harboring pACYC-184-EGFP, and plated to LB agar plate containing Cm (17 μg mL^{-1}). The transformants were transformed again with the same protocol as above with pEK-bhr-antiEGFP.

To analyze EGFP fluorescence from *C. necator* (Fig. 2b), the strains were inoculated to 2 mL LB media in 14 mL test tubes supplemented with appropriate antibiotics and 1 mM of IPTG to induce the expression of the sRNA system, then incubated at 30°C, with rotary shaking at 200 rpm overnight. The cells were grown for 24 h. The cells were harvested by centrifugation at 4,000×g, washed with PBS, and resuspended to PBS. Subsequently, the resulting samples were transferred to a black, clear-bottom 96-well plate, and OD_{600} and fluorescence intensities were measured using Spark Multimode Microplate Reader.

Knockdown of reporter genes in *Lactococcus lactis*

Transformation of *L. lactis* with *L. lactis–E. coli* shuttle vectors were performed by electroporation [25]. To analyze sRNA-mediated knockdown of the target gene in *L. lactis* (Fig. 2b), overnight cultures of *L. lactis* strains harboring *upp*-targeting sRNA plasmids were inoculated to 14 mL test tubes containing 2 mL BHIS medium supplemented with 5 μg mL^{-1} tetracycline and 500 μg mL^{-1} 5-fluorouracil. The cells were cultured in 30 °C incubators without shaking for 24 h, and cell growth was determined by measuring OD_{600}.

Knockdown of reporter genes in *Streptomyces coelicolor*

Transformation of *S. coelicolor* with *Streptomyces–E. coli* shuttle vectors was performed by conjugation[26]. To test the production of actinorhodin in *S. coelicolor* strains harboring sRNAs (Fig.

2b), *S. coelicolor* exconjuvants confirmed to contain either of pGM-CTRL or pGMbhr-anti-actIORFI were first spread onto MS agar plate supplemented with 10 mM of $MgCl_2$, 50 μg mL^{-1} apramycin, and 30 μg mL^{-1} nalidixic acid. After incubation for 5 d at 30°C, the spores were collected as described above, centrifugation at 5,000×*g* for 5 min and were resuspended in 2 mL of water. The OD_{600} of the spore suspensions was adjusted to 1, and 200 μL of the diluted spore suspension was spread onto MS agar plates supplemented with 10 mM of $MgCl_2$, 50 μg mL^{-1} apramycin, 30 μg mL^{-1} nalidixic acid, and 5 μg mL^{-1} thiostrepton prepared on 35×10 mm petri dishes. After incubation for 1 week, photos of the plates were taken. To quantify actinorhodin produced from the agar plates, the agar containing actinorhodin was dissolved in hot water and pH was adjusted to ~2. After extraction with equal volume of chloroform, the chloroform layer was isolated and was dried using nitrogen evaporator, which was resolubilized by 1 mL of methanol. Actinorhodin was quantified by measuring the absorbance of the extracted samples at 530 nm with Ultrospec 3100 spectrophotometer (Amerhsam Biosciences, Uppsala, Sweden).

Knockdown of reporter genes in *Staphylococcus epidermidis*

Transformation of *S. epidermidis* with *S. aureus–E. coli* shuttle vectors was performed by electroporation[27], with minor modifications. Briefly, *S. epidermidis* colonies grown on BHI agar plates were inoculated to 10 mL of TSB (BD Difco) medium and were cultured at 37°C, 200 rpm overnight. Then, the cells were diluted to OD_{600} of 0.5 in 300 mL Erlenmeyer flasks each containing 100 mL of TSB, and were additionally grown at 37°C, 200 rpm for 30 min (until OD_{600} reaches 0.8~0.9). After chilling the cells in ice for 10 min, the cells were collected by centrifugation at 5,000×*g* for 5 min and were resuspended in 20 mL of cold 10% (v/v) glycerol. This washing step was repeated one more time, and the cells were resuspended in 250 μL of 10% glycerol. Then, 50 μL aliquots were flash frozen in liquid nitrogen, and were stored at -70°C until use. For electroporation, the competent cells were thawed on ice, and were left at room temperature for 5 min. The cells were collected by centrifugation at 7,000×*g* for 2 min and were resuspended in 50 μL of electroporation buffer (10% glycerol, 500 mM sucrose). Then, 5 μg of plasmid prepared from *E. coli* ET2925 (Dcm^- Dam^-) was added and the mixture was incubated at room temperature for 10 min. Electroporation was performed with the following conditions: 21 kV cm^{-1}, 100 ohm, 25 μF. After electroporation, the cells were recovered in 1 mL of TSB containing 500 mM sucrose at 37°C for 1 h, and were plated on BHI agar containing appropriate antibiotics [5 μg mL^{-1} Cm or

15 μg mL^{-1} neomycin (Neo)]. The recombinant plasmid pSK9065 was first transformed to *S. epidermidis*, and plated to BHI agar plate containing 15 μg mL^{-1} of Neo. The transformants were transformed again with the same protocol as above with pRMCbhr-anti-mRFPmars.

To analyze RFP fluorescence from *S. epidermidis* (Fig. 2b), the strains were inoculated to 5 mL TSB media in 50 mL falcon tubes supplemented with appropriate antibiotics. After overnight culture at 37°C, 200 rpm, the cells were transferred (2% volumetric ratio) to different 50 mL falcon tubes containing 5 mL TSB media supplemented with appropriate antibiotics and 1 μg mL^{-1} of anhydrotetracycline. The cells were grown for 24 h at 37°C, 200 rpm. The cells were harvested by centrifugation at 5,000×*g*, washed with PBS, and resuspended to PBS. Subsequently, the resulting samples were transferred to a black, clear-bottom 96-well plate, and OD_{600} and fluorescence intensities were measured using Spark Multimode Microplate Reader.

Knockdown of reporter genes in *Pseudomonas putida*, *Escherichia coli*, and *Chromobacterium violaceum*

Transformation of *P. putida*, *E. coli* DH5α, *E. coli* Nissle 1917, and *C. violaceum* with the corresponding sRNA platform plasmids were performed by electroporation following the basic protocols as reported[23]. For *P. putida*, KT2440 ΔpvdD1k (ref. [28]) was used as the control strain without a reporter. KT2440 ΔpvdD::EGFP (ref. [28]) with *EGFP* integrated in the genome was used as another control strain. KT2440 ΔpvdD::EGFP was transformed with pBBR1bhr-anti-EGFP. For *C. violaceum*, pBBR1bhr-anti-vioA was transformed to the wild type *C. violacein* strain. For the *E. coli* strains, pACYC-EGFP reporter plasmid was first introduced, followed by transformation of pEKbhr-anti-EGFP.

To test the knockdown of reporters (Fig. 2b), the strains were inoculated to 5 mL LB media in test tubes supplemented with appropriate antibiotics. After overnight culture at 30°C, 200 rpm, the cells were transferred (2% volumetric ratio) to different test tubes containing 5 mL LB media supplemented with appropriate antibiotics and 1 mM of IPTG to induce the sRNA system. The cells were grown for 24 h at 30°C. For *P. putida*, *E. coli* DH5α, and *E. coli* Nissle 1917, EGFP was employed as the reporter. The cells were harvested by centrifugation at 10,000×*g* for 1 min, washed and resuspended with the sample volume of PBS. Subsequently, the resulting samples

were transferred to a black, clear-bottom 96-well plate, and OD_{600} and fluorescence intensities were measured using Spark Multimode Microplate Reader. For *C. violaceum*, 100 μL of the culture was extracted with 900 μL of DMSO, vortexed vigorously for 1 min, and centrifuged at 10,000×*g* for 1 min. The supernatant samples were filtered through 0.2 μm PTFE syringe filters and the concentrations of violacein and deoxyviolacein were analyzed using HPLC (details described in the 'Analytical methods' section below).

Knockdown of reporter genes in *Vibrio natriegens*

Transformation of *V. natriegens* with plasmids was performed by electroporation[29], with slight modification. Briefly, *V. natriegens* colonies grown on LB agar plates were inoculated to 10 mL of LB medium and were cultured at 37°C, 200 rpm overnight. Then, the cells were transferred (2% volumetric ratio) to 300 mL Erlenmeyer flasks each containing 100 mL of LB, and were additionally grown at 37°C, 200 rpm until OD_{600} reaches 0.5. After chilling the cells in ice for 10 min, the cells were collected by centrifugation at 5,000×*g* for 5 min and were resuspended in 20 mL of cold electroporation buffer (680 mM sucrose, 7 mM K_2HPO_4, pH 7). This washing step was repeated one more time, and the cells were resuspended in about 3 mL of the electroporation buffer. Then, 100 μL aliquots were flash frozen in liquid nitrogen, and were stored at -70°C until use. Electroporation was performed with the following conditions: 8 kV cm^{-1}, 200 ohm, 25 μF. After electroporation, the cells were recovered in 1 mL of LB containing 680 mM sucrose at 37°C for 1 h, and were plated on LB agar containing appropriate antibiotics. The reporter pACYC-EGFP plasmid was first transformed, followed by transformation of pEKbhr-anti-EGFP. Analysis of EGFP fluorescence was performed using the same protocol as described above for *E. coli*.

Supplementary Method 3. Construction of engineered *C. glutamicum* strains

To construct the single-base pair exchange vectors, the upstream and downstream DNA regions flanking the target single-base pairs were amplified by PCR using primers pairs MANT-KD1-F1_F/MANT-KD1-F1_R and MANT-KD1-F2_F/MANT-KD1-F2_R for *gnd*, MANT-KD2-F1_F/MANT-KD2-F1_R and MANT-KD2-F2_F/MANT-KD2-F2_R for *pgl*, and MANT-KD3-F1_F/MANT-KD3-F1_R and MANT-KD3-F2_F/MANT-KD3-F2_R for *tkt* from the *C. glutamicum* DBDH genomic DNA and using primer pairs IND-KD1-F1_F/IND-KD1-F1_R and

IND-KD1-F2_F/IND-KD1-F2_R for *NCgl2113*, IND-KD2-F1_F/IND-KD2-F1_R and IND-KD2-F2_F/IND-KD2-F2_R for *ruvA*, IND-KD3-F1_F/IND-KD3-F1_R and IND-KD3-F2_F/IND-KD3-F2_R for *NCgl0549*, IND-KD4-F1_F/IND-KD4-F1_R and IND-KD4-F2_F/IND-KD4-F2_R for *purC*, IND-KD5-F1_F/IND-KD5-F1_R and IND-KD5-F2_F/IND-KD5-F2_R for *NCgl2907* from the *C. glutamicum* BIRU-NP genomic DNA (Supplementary Data 6). The amplified upstream and downstream DNA fragments were combined to make single linear DNA fragments with the exchanged codon situated between the upstream and downstream region by overlap extension PCR with primer pairs MANT-KD1-F1_F/MANT-KD1-F2_R, MANT-KD2-F1_F/MANT-KD2-F2_R, MANT-KD3-F1_F/MANT-KD3-F2_R, IND-KD1-F1_F/IND-KD1-F2_R, IND-KD2-F1_F/IND-KD2-F2_R, IND-KD3-F1_F/IND-KD3-F2_R, IND-KD4-F1_F/IND-KD4-F2_R, and IND-KD5-F1_F/IND-KD5-F2_R for genes *gnd*, *pgl*, *tkt*, *NCgl2113*, *ruvA*, *NCgl0549*, *purC*, and *NCgl2907* respectively. These fragments were then individually cloned to PstI and BamHI double-digested pK19mobsacB vector to yield pK19msb-MKD1, pK19msb-MKD2, pK19msb-MKD3, pK19msb-IKD1, pK19msb-IKD2, pK19msb-IKD3, pK19msb-IKD4, and pK19msb-IKD5, respectively (Supplementary Data 5).

Addition of 6×His(histidine) tags to the 3’-end of target genes was performed in the same manner. Briefly, the upstream and downstream DNA regions flanking genes were amplified by PCR using primers pairs INDhis_LA_F/INDhis_LA_R and INDhis_RA_F/INDhis_RA_R for *ruvA* from the *C. glutamicum* BIRU-NP genomic DNA and using primer pairs VLhis_gdh_LA_F/VLhis_gdh_LA_R and VLhis_gdh_RA_F/VLhis_gdh_RA_R for *gdh*, VLhis_hom_LA_F/VLhis_hom_LA_R and VLhis_hom_RA_F/VLhis_hom_RA_R for *hom*, and VLhis_icd_LA_F/VLhis_icd_LA_R and VLhis_icd_RA_F/VLhis_icd_RA_R for *icd* from the *C. glutamicum* VL2 genomic DNA. The amplified upstream and downstream DNA were combined by overlap extension PCR with primer pairs INDhis_LA_F/INDhis_RA_R for *ruvA*, VLhis_gdh_LA_F/VLhis_gdh_RA_R for *gdh*, VLhis_hom_LA_F/VLhis_hom_RA_R for *hom*, and VLhis_icd_LA_F/VLhis_icd_RA_R for *icd*. The fragments were then individually cloned to PstI and BamHI double-digested pK19mobsacB vector to yield pK19msb-INDhis, pK19msb-VLhis1, pK19msb-VLhis2, and pK19msb-VLhis3.

Single base-pair changes and addition of histidine tags for *C. glutamicum* were done using a *sacB*-based genetic engineering method[30]. Cells were transformed with one of the pK19msb-series vectors-pK19msb-MKD1, pK19msb-MKD2, pK19msb-MKD3, pK19msb-IND1, pK19msb-IND2, pK19msb-IND3, pK19msb-IND4, pK19msb-IND5, pK19msb-INDhis, pK19msb-VLhis1, pK19msb-VLhis2, and pK19msb-VLhis3 (Supplementary Data 5)—by electroporation and plated onto BHIS plate containing Km. After incubating the cells for 48 h in 30°C, three single colonies were chosen from the plate and were each inoculated to 15 mL test tubes each containing 2 mL of BHIS medium and cultured in a 30°C shaking incubator at 200 rpm for 12 h. The cells were then diluted 1,000 times and spread onto BHIS agar plates containing 14%(w/v) sucrose and incubated for another 48 h in 30°C. The resulting colonies that grew on the sucrose containing BHIS agar plates were selected for loss of Km resistance and were confirmed of the genetic manipulation with PCR or sequencing. Strains MANTKD1, MANTKD2, MANTKD3, BIRU12, BIRU13, BIRU14, BIRU15, and BIRU16 were generated with the pK19msb-series vectors using the abovementioned *sacB*-based genetic engineering method. Strains BIRU17, BIRU18, and BIRU19 were constructed from BIRU12 and BIRU16 using the corresponding pK19msb-series vectors, and strain BIRU20 was constructed from BIRU19 using the pK19msb-IND1. Strains BIRU-his-ruvA and BIRU20-his-ruvA were constructed using pK19msb-INDhis. The VL2-His strain was constructed using pK19msb-VLhis1, pK19msb-VLhis2, and pK19msb-VLhis3.

Supplementary Note 1. Employing a two-plasmid system for testing sRNA-mediated target gene knockdown

Although a previous study reported that employing the MicC and *E. coli* Hfq-based sRNA system can efficiently knockdown target genes in *C. glutamicum*[11], several artifacts might have hindered obtaining precise data. First, using a single plasmid for the expression of a reporter gene as well as an anti-reporter sRNA might mask the outcome by directly affecting the expression of the reporter gene, aside from the effect endowed by the sRNA. An example of a downstream gene repressing the expression of an upstream gene has been previously reported[17]. Second, previous studies used weak promoters to express reporter genes, which would make the knockdown efficiency to be seen increased. For these reasons, we sought to employ a two-plasmid system (one plasmid with the reporter gene and another plasmid with the sRNA system) with a strong constitutive promoter (I16) for the reporter gene to test the knockdown efficiency of the sRNA system in *C. glutamicum*. This would allow a more stringent condition for testing the knockdown efficiency as well as allowing orthogonal expression of the reporter and the sRNA.

Supplementary Note 2. Comparing the knockdown efficiency of the BHR-sRNA system with that of CRISPRi

The BHR-sRNA system was compared to the CRISPRi system, one of the standard techniques for bacterial target gene knockdown; the CRISPRi system demonstrated a similar knockdown efficiency (65.4%) compared with that (65.2%) by the BHR-sRNA system (*P* = 0.79, Supplementary Fig. 2a). While the final OD_{600} of the BHR-sRNA harboring strain remained unchanged when compared with that of the control strain only expressing *GFP* (*P* = 0.76), the final OD_{600} of the CRISPRi-harboring strain was decreased by 16.4% (*P* = 0.0052) when compared with that of the control strain. This was possibly due to the increased metabolic burden by the large-sized dCas9 protein (~160 kDa). By contrast, the size of BsHfq protein (24.6 kDa) is much smaller than that of dCas9, resulting in normal cell growth (Supplementary Fig. 2a).

In addition, the transcript levels of GFP were retained when using the BHR-sRNA system while 35.4 % decrease in GFP transcript level was observed using the CRISPRi system (*P* = 0.047; Supplementary Fig. 2c), indicating that sRNA represses genes at the translational level unlike CRISPRi which represses genes at the transcriptional level.

Supplementary Note 3. Knockdown of metabolic genes by the BHR-sRNA system

To construct industrially relevant bacterial strains, assessing the changes in the level of metabolites produced as an outcome of repressing a target gene is important. As the first target, a type III polyketide synthase RppA from *Streptomyces griseus* was selected. RppA converts five molecules of malonyl-CoA into a red-colored pigment flaviolin, without the aid of any other auxiliary enzymes or cofactors (Supplementary Fig. 3b). Thus, RppA is a good target which was also identified to be consistent in multiple bacterial species[17]. As a result, we found that the BHR-sRNA system successfully reduced the production of flaviolin by 64.3% in *R. opacus* and 84.1% in *E. coli* DH5α (Supplementary Fig. 3c,d).

Next, to assess the performance of the BHR-sRNA platform in *C. glutamicum* for metabolic engineering applications, the effect of BHR-sRNA-mediated knockdown of genes on metabolite production was investigated. For this purpose, two knockdown gene targets were chosen. First, the *lysA* gene encoding diaminopimelate decarboxylase in a L-lysine-overproducing *C. glutamicum* strain BE was targeted[30] (Supplementary Fig. 3e). Since *lysA* was responsible for the last step of L-lysine biosynthesis (conversion of *meso*-2,6-diaminoheptanedioate into L-lysine), knockdown of *lysA* was expected to reduce L-lysine production. Shake flask cultivation of the strain harboring the anti-*lysA* sRNA showed 20.7% decrease in L-lysine production when compared with that from the strain without sRNA ($P = 2.0 \times 10^{-4}$; Supplementary Fig. 3f), which was in agreement with the previous report[31]. Although the repression of L-lysine was less than anticipated, it must be noted that there might be other isozymes of diaminopimelate decarboxylase or unknown regulatory mechanisms on L-lysine production in the BE strain. The second target chosen was the *pyc* gene encoding pyruvate carboxylase, a target known to repress growth on sodium lactate when knocked-down[32] (Supplementary Fig. 3g). When grown on 20 g L^{-1} sodium lactate as the sole carbon source in CGXII minimal medium, the maximum growth (OD_{600}) of the *C. glutamicum* strain harboring the anti-*pyc* sRNA was reduced to 82.9% ($P = 1.2 \times 10^{-6}$) compared with that of wild-type *C. glutamicum* harboring pEKEx1 after 24 h shake flask cultivation (Supplementary Fig. 3h). This phenotype has been shown consistently in *pyc* deleted or repressed

strains[32,33], demonstrating that the sRNA platform developed here can efficiently regulate the metabolic fluxes and is thus suitable for metabolic engineering purposes.

Supplementary Note 4. Rapid identification of gene knockdown targets for enhanced production of MANT

We applied the BHR-sRNA system to screen gene knockdown targets for enhanced production of methyl anthranilate (MANT) in *C. glutamicum*. MANT is an aromatic compound that gives grape flavoring and scent, which serves as an important ingredient in perfumes and cosmetics. We have previously developed a metabolically engineered *C. glutamicum* strain capable of producing large amounts of bio-based MANT through fermentation[34]. While the MANT producing strain has already been extensively engineered, we reasoned that identification of additional gene knockdown targets can be done using *in silico* genome-scale metabolic analyses together with the sRNA system (Supplementary Fig. 6a).

To identify gene knockdown targets for enhanced production of MANT, two different algorithms, minimization of metabolic adjustment (MOMA) and flux scanning based on enforced objective flux (FSEOF) were employed. *C. glutamicum* genome-scale metabolic model iCW773 (ref. [35]) was contextualized for MANT production, where *qsuB* and *qsuD* genes were removed, *aamt1* added, with *aroG*^{S180F} and *sahH* genes overexpressed[34]. Throughout the simulation, the glucose uptake rate was set at 10 mmol gDCW^{-1} h^{-1}. All simulations were conducted in Python environment using GurobiPy package (Gurobi Optimization, Inc., Houston, TX). Reading, writing, and manipulation of the COBRA-compliant SBML files were implemented using COBRApy[36]. Thus, 15 gene targets were selected from flux balance analysis using MOMA (Supplementary Data 2), FSEOF (Supplementary Data 3), and from previous literature (Supplementary Fig. 6b, Supplementary Table 7).

The sRNA plasmids pEK-bhr-anti-1513 through pEK-bhr-anti-2200 were constructed and introduced to the MANT-producing *C. glutamicum* strain DBDH/pCS-amGH to construct strains MANT-S1 through MANT-S15 (Supplementary Data 5). In shake flask cultures, the strains MANT-S2, MANT-S7, and MANT-S5 targeting genes *gnd* (*NCgl1396*) encoding 6-phosphogluconate dehydrogenase, *tkt* (*NCgl1512*) encoding transketolase, and *pgl* (*NCgl1516*) encoding a putative 6-phosphogluconolactonase showed increased MANT titers compared to that from the control strain DBDH/pCS-amGH (Supplementary Fig. 6c). Taking a step further, to eliminate sRNA-harboring plasmids, the start codon of the three target genes were changed from

ATG to GTG to construct the strains MANTKD1, MANTKD2, and MANTKD3 (Supplementary Data 5). Shake flask cultivation showed that the knockdown of *gnd* (*NCgl1396*) resulted in 223 mg L^{-1} of MANT production, which corresponds to 16% increase when compared with that from the control strain (P = 0.040; Supplementary Fig. 6e). The strategy showcased here demonstrates how prototyping using the BHR-sRNA system enables a quick and streamlined method for screening and selecting metabolic engineering targets.

Supplementary Note 5. Abbreviations used in Figure 3b and Figure 3e

Abbreviations: 5AVA, 5-aminovalerate; 5AVA-CoA, 5-aminovalerate-CoA; 5AVM, 5-aminovaleramide; Ac-CoA, acetyl-coA; ACT, acetate; AKG, α-ketoglutarate; ASA, aspartate semialdehyde; ASP, aspartate; CIT, citrate; DAHP, 3-deoxy-D-arabinoheptulosonate 7-phosphate; DHAP, dihydroxyacetonephosphate; E4P, erythrose 4-phosphate; F1,6-P, fructose-1,6-biphosphate; FUM, fumarate; G3P glyceraldehyde-3-phosphate; G6P, glucose 6-phosphate; GLC, glucose; GTA, glutaric acid; ICA, isocitrate; IND, indigoidine; GLN, L-glutamine; GLX, glyoxylate; GLU, L-glutamate; Glu^{tRNA}, glutamyl-tRNA; LYS, L-lysine; MAL, malate; M-DAP, *meso*-diaminopimelate; OAA, oxaloacetate; PEP, phosphoenolpyruvate; PG, peptidoglycan; PPP, pentose phosphate pathway, PYR, pyruvate; SUC, succinate; SUC-CoA, succinyl-CoA; THR, l-threonine; VLM, valerolactam.

Supplementary references

1. Na, D. et al. Metabolic engineering of *Escherichia coli* using synthetic small regulatory RNAs. *Nat. Biotechnol.* **31**, 170-174 (2013).
2. Kim, B., Park, H., Na, D. & Lee, S.Y. Metabolic engineering of *Escherichia coli* for the production of phenol from glucose. *Biotechnol. J.* **9**, 621-629 (2014).
3. Chae, T.U., Kim, W.J., Choi, S., Park, S.J. & Lee, S.Y. Metabolic engineering of *Escherichia coli* for the production of 1,3-diaminopropane, a three carbon diamine. *Sci. Rep.* **5**, 13040 (2015).
4. Yang, D. et al. Expanded synthetic small regulatory RNA expression platforms for rapid and multiplex gene expression knockdown. *Metab. Eng.* **54**, 180-190 (2019).
5. Zhang, J. et al. Synthetic sRNA-based engineering of *Escherichia coli* for enhanced Production of full-length immunoglobulin G. *Biotechnol. J.* **15**, 1900363 (2020).
6. Noh, M., Yoo, S.M., Yang, D. & Lee, S.Y. Broad-spectrum gene repression using scaffold engineering of synthetic sRNAs. *ACS Synth. Biol.* **8**, 1452-1461 (2019).
7. Cao, Y., Li, X., Li, F. & Song, H. CRISPRi-sRNA: Transcriptional-translational regulation of extracellular electron transfer in *Shewanella oneidensis*. *ACS Synth. Biol.* **6**, 1679-1690 (2017).
8. Apura, P. et al. Tailor-made sRNAs: a plasmid tool to control the expression of target mRNAs in *Pseudomonas putida*. *Plasmid* **109**, 102503 (2020).
9. Liu, Y. et al. Modular pathway engineering of *Bacillus subtilis* for improved *N*-acetylglucosamine production. *Metab. Eng.* **23**, 42-52 (2014).
10. Cho, C. & Lee, S.Y. Efficient gene knockdown in *Clostridium acetobutylicum* by synthetic small regulatory RNAs. *Biotechnol. Bioeng.* **114**, 374-383 (2017).
11. Sun, D. et al. Metabolic engineering of *Corynebacterium glutamicum* by synthetic small regulatory RNAs. *J. Ind. Microbiol. Biotechnol.* **46**, 203-208 (2019).
12. Sun, T. et al. Re-direction of carbon flux to key precursor malonyl-CoA via artificial small RNAs in photosynthetic *Synechocystis* sp. PCC 6803. *Biotechnol. Biofuels* **11**, 26 (2018).
13. Li, S., Sun, T., Xu, C., Chen, L. & Zhang, W. Development and optimization of genetic toolboxes for a fast-growing cyanobacterium *Synechococcus elongatus* UTEX 2973. *Metab. Eng.* **48**, 163-174 (2018).

14. Wang, L.-J., Jiang, X.-R., Hou, J., Wang, C.-H. & Chen, G.-Q. Engineering *Halomonas bluephagenesis* via small regulatory RNAs. *Metab. Eng.* **73**, 58-69 (2022).
15. Ghiffary, M.R. et al. High-level production of the natural blue pigment indigoidine from metabolically engineered *Corynebacterium glutamicum* for sustainable fabric dyes. *ACS Sustain. Chem. Eng.* **9**, 6613-6622 (2021).
16. Gibson, D.G. et al. Enzymatic assembly of DNA molecules up to several hundred kilobases. *Nat. Methods* **6**, 343-345 (2009).
17. Cho, J.S. et al. CRISPR/Cas9-coupled recombineering for metabolic engineering of *Corynebacterium glutamicum*. *Metab. Eng.* **42**, 157-167 (2017).
18. So, Y. et al. A highly efficient CRISPR-Cas9-mediated large genomic deletion in *Bacillus subtilis*. *Front. Microbiol.* **8**, 1167 (2017).
19. Kim, H.M., Chae, T.U., Choi, S.Y., Kim, W.J. & Lee, S.Y. Engineering of an oleaginous bacterium for the production of fatty acids and fuels. *Nat. Chem. Biol.* **15**, 721-729 (2019).
20. Tong, Y., Charusanti, P., Zhang, L., Weber, T. & Lee, S.Y. CRISPR-Cas9 based engineering of actinomycetal genomes. *ACS Synth. Biol.* **4**, 1020-1029 (2015).
21. Corrigan, R.M. & Foster, T.J. An improved tetracycline-inducible expression vector for *Staphylococcus aureus*. *Plasmid* **61**, 126-129 (2009).
22. Kovach, M.E. et al. Four new derivatives of the broad-host-range cloning vector pBBR1MCS, carrying different antibiotic-resistance cassettes. *Gene* **166**, 175-176 (1995).
23. Sambrook, J. & Russell, D.W. Molecular cloning: a laboratory manual 3rd edition. (Cold Spring Harbor Laboratory Press, New York; 2001).
24. Han, J., Qiu, Y.Z., Liu, D.C. & Chen, G.Q. Engineered *Aeromonas hydrophila* for enhanced production of poly(3-hydroxybutyrate-co-3-hydroxyhexanoate) with alterable monomers composition. *FEMS Microbiol. Lett.* **239**, 195-201 (2004).
25. Holo, H. & Nes, I.F. High-frequency transformation, by electroporation, of *Lactococcus lactis* subsp. *cremoris* grown with glycine in osmotically stabilized media. *Appl. Environ. Microbiol.* **55**, 3119-3123 (1989).
26. Tong, Y. et al. CRISPR-Cas9, CRISPRi and CRISPR-BEST-mediated genetic manipulation in streptomycetes. *Nat. Protoc.* **15**, 2470-2502 (2020).

27. Lofblom, J., Kronqvist, N., Uhlen, M., Stahl, S. & Wernerus, H. Optimization of electroporation-mediated transformation: *Staphylococcus carnosus* as model organism. *J. Appl. Microbiol.* **102**, 736-747 (2007).
28. Choi, K.R., Cho, J.S., Cho, I.J., Park, D. & Lee, S.Y. Markerless gene knockout and integration to express heterologous biosynthetic gene clusters in *Pseudomonas putida*. *Metab. Eng.* **47**, 463-474 (2018).
29. Weinstock, M.T., Hesek, E.D., Wilson, C.M. & Gibson, D.G. *Vibrio natriegens* as a fast-growing host for molecular biology. *Nat. Methods* **13**, 849-851 (2016).
30. Han, T.H., Kim, G.B. & Lee, S.Y. Glutaric acid production by systems metabolic engineering of an L-lysine–overproducing *Corynebacterium glutamicum*. *Proc. Natl. Acad. Sci. USA* **117**, 30328-30334 (2020).
31. Kind, S. & Wittmann, C. Bio-based production of the platform chemical 1,5-diaminopentane. *Appl. Microbiol. Biotechnol.* **91**, 1287-1296 (2011).
32. Park, J., Shin, H., Lee, S.M., Um, Y. & Woo, H.M. RNA-guided single/double gene repressions in *Corynebacterium glutamicum* using an efficient CRISPR interference and its application to industrial strain. *Microb. Cell Fact.* **17**, 4 (2018).
33. Peters-Wendisch, P.G. et al. Pyruvate carboxylase from *Corynebacterium glutamicum*: characterization, expression and inactivation of the pyc gene. *Microbiology* **144 (Pt 4)**, 915-927 (1998).
34. Luo, Z.W., Cho, J.S. & Lee, S.Y. Microbial production of methyl anthranilate, a grape flavor compound. *Proc. Natl. Acad. Sci. U S A* **116**, 10749-10756 (2019).
35. Zhang, Y. et al. A new genome-scale metabolic model of *Corynebacterium glutamicum* and its application. *Biotechnol. Biofuels* **10**, 169 (2017).
36. Ebrahim, A., Lerman, J.A., Palsson, B.O. & Hyduke, D.R. COBRApy: constraints-based reconstruction and analysis for Python. *BMC Syst. Biol.* **7**, 74 (2013).
