# Supplementary material for: Targeted and high-throughput gene knockdown in diverse bacteria using synthetic sRNAs
Source: Nat Commun. 2023 Apr 24;14:2359. doi: 10.1038/s41467-023-38119-y (PMC10126203; doi:10.1038/s41467-023-38119-y)
Supplement: Supplementary file 3 — Description of Additional Supplementary Files [file 41467_2023_38119_MOESM3_ESM.pdf]

Description of Additional Supplementary Files

File Name: Supplementary Data 1
Description: Literature mining results for the screening of Gram-positive sRNA systems

File Name: Supplementary Data 2
Description: Flux balance analysis using MOMA for enhanced production of MANT in *C. glutamicum*

File Name: Supplementary Data 3
Description: Flux balance analysis using FSEOF for enhanced production of MANT in *C. glutamicum*

File Name: Supplementary Data 4
Description: Genome-wide BHRsRNA pooled library sequence covering 2,959 *C. glutamicum* genes

File Name: Supplementary Data 5
Description: Strains and plasmids used in this study

File Name: Supplementary Data 6
Description: Oligonucleotides used in this study
