# Supplementary material for: Targeted and high-throughput gene knockdown in diverse bacteria using synthetic sRNAs
Source: Nat Commun. 2023 Apr 24;14:2359. doi: 10.1038/s41467-023-38119-y (PMC10126203; doi:10.1038/s41467-023-38119-y)
Supplement: Supplementary file 10 — Reporting Summary [file 41467_2023_38119_MOESM10_ESM.pdf]

Corresponding author(s): Sang Yup Lee
Last updated by author(s): Apr 10, 2023

# Reporting Summary

Nature Portfolio wishes to improve the reproducibility of the work that we publish. This form provides structure for consistency and transparency in reporting. For further information on Nature Portfolio policies, see our Editorial Policies and the Editorial Policy Checklist.

## Statistics

For all statistical analyses, confirm that the following items are present in the figure legend, table legend, main text, or Methods section.

| n/a | Confirmed | |
|---|---|---|
| ☐ | ☒ | The exact sample size (*n*) for each experimental group/condition, given as a discrete number and unit of measurement |
| ☐ | ☒ | A statement on whether measurements were taken from distinct samples or whether the same sample was measured repeatedly |
| ☐ | ☒ | The statistical test(s) used AND whether they are one- or two-sided<br>*Only common tests should be described solely by name; describe more complex techniques in the Methods section.* |
| ☒ | ☐ | A description of all covariates tested |
| ☐ | ☒ | A description of any assumptions or corrections, such as tests of normality and adjustment for multiple comparisons |
| ☐ | ☒ | A full description of the statistical parameters including central tendency (e.g. means) or other basic estimates (e.g. regression coefficient) AND variation (e.g. standard deviation) or associated estimates of uncertainty (e.g. confidence intervals) |
| ☐ | ☒ | For null hypothesis testing, the test statistic (e.g. $F$, $t$, $r$) with confidence intervals, effect sizes, degrees of freedom and $P$ value noted<br>*Give P values as exact values whenever suitable.* |
| ☒ | ☐ | For Bayesian analysis, information on the choice of priors and Markov chain Monte Carlo settings |
| ☒ | ☐ | For hierarchical and complex designs, identification of the appropriate level for tests and full reporting of outcomes |
| ☒ | ☐ | Estimates of effect sizes (e.g. Cohen's *d*, Pearson's *r*), indicating how they were calculated |

*Our web collection on statistics for biologists contains articles on many of the points above.*

## Software and code

Policy information about availability of computer code

| | |
|---|---|
| Data collection | No software was used |
| Data analysis | LightCycler(R) 96 (Roche) version 1.1.0.1320 was used for analyzing RT-PCR results. Image Lab (BioRad) version 6.0.0 was used for measuring the intensity of western-blot and Coomassie-stained SDS-PAGE bands. Phylogenetic tree was generated using phyloT (https://phylot.biobyte.de/) and visualized with iTOL. FlowJo version 10.0.8 and BDFACS Diva version 6.2 was used to visualize FACS histogram. To identify knockdown gene targets for enhanced production of MANT, two different algorithms, minimization of metabolic adjustment (MOMA) and flux scanning based on enforced objective flux (FSEOF) were used. |

For manuscripts utilizing custom algorithms or software that are central to the research but not yet described in published literature, software must be made available to editors and reviewers. We strongly encourage code deposition in a community repository (e.g. GitHub). See the Nature Portfolio guidelines for submitting code & software for further information.

# Data

Policy information about availability of data

All manuscripts must include a data availability statement. This statement should provide the following information, where applicable:

- Accession codes, unique identifiers, or web links for publicly available datasets
- A description of any restrictions on data availability
- For clinical datasets or third party data, please ensure that the statement adheres to our policy

Source data for all the datasets of this study are provided with this paper.

# Research involving human participants, their data, or biological material

Policy information about studies with human participants or human data. See also policy information about sex, gender (identity/presentation), and sexual orientation and race, ethnicity and racism.

| | |
|---|---|
| Reporting on sex and gender | Not applicable |
| Reporting on race, ethnicity, or other socially relevant groupings | Not applicable |
| Population characteristics | Not applicable |
| Recruitment | Not applicable |
| Ethics oversight | Not applicable |

Note that full information on the approval of the study protocol must also be provided in the manuscript.

# Field-specific reporting

Please select the one below that is the best fit for your research. If you are not sure, read the appropriate sections before making your selection.

☒ Life sciences ☐ Behavioural & social sciences ☐ Ecological, evolutionary & environmental sciences

For a reference copy of the document with all sections, see nature.com/documents/nr-reporting-summary-flat.pdf

# Life sciences study design

All studies must disclose on these points even when the disclosure is negative.

| | |
|---|---|
| Sample size | Most experiments were done in triplicates, which is a normally employed sample size to ensure biological reproducibility, unless otherwise specified. For flask culture presented in Extended Data Fig. 4c, experiments were performed in duplicates as the key result of this experiments were also confirmed by flask cultivation of the resulting genome engineered strains. Reference papers for the determination of biological sample sizes are as follows: PMID 35190685; PMID 31209347. |
| Data exclusions | No data were excluded. |
| Replication | Experimental findings were reproduced by duplicates or triplicates. Fed-batch fermentation was repeated for reproducibility. |
| Randomization | All colonies were randomly selected from plates containing about 100–200 colonies and subject to independent flask culture and chemical analysis. As colonies with similar size and appearance were randomly chosen, experimental group allocation was performed randomly. |
| Blinding | The investigators were blinded to the group allocation by randomly selecting single colonies multiple times. |

# Reporting for specific materials, systems and methods

We require information from authors about some types of materials, experimental systems and methods used in many studies. Here, indicate whether each material, system or method listed is relevant to your study. If you are not sure if a list item applies to your research, read the appropriate section before selecting a response.

## Materials & experimental systems

| n/a | Involved in the study |
| --- | --- |
| ☐ | ☒ Antibodies |
| ☒ | ☐ Eukaryotic cell lines |
| ☒ | ☐ Palaeontology and archaeology |
| ☒ | ☐ Animals and other organisms |
| ☒ | ☐ Clinical data |
| ☒ | ☐ Dual use research of concern |
| ☒ | ☐ Plants |

## Methods

| n/a | Involved in the study |
| --- | --- |
| ☒ | ☐ ChIP-seq |
| ☐ | ☒ Flow cytometry |
| ☒ | ☐ MRI-based neuroimaging |

# Antibodies

| | |
| --- | --- |
| Antibodies used | Anti-6X His tag antibody (HRP), Abcam, Cat# ab1187; RRID: AB_298652 |
| Validation | HRP Anti-6X His tag® antibody (ab1187): Validated by the manufacturer and multiple publications. e.g., Madrid R et al., Foods 10 (2021), PMID 33671390; Cho G et al., Sci Rep 11:5785 (2021), PMID 33707636; Hershewe JM et al., Nat Commun 12:2363 (2021), PMID 33888690. |

# Flow Cytometry

## Plots

Confirm that:

☒ The axis labels state the marker and fluorochrome used (e.g. CD4-FITC).

☒ The axis scales are clearly visible. Include numbers along axes only for bottom left plot of group (a 'group' is an analysis of identical markers).

☒ All plots are contour plots with outliers or pseudocolor plots.

☒ A numerical value for number of cells or percentage (with statistics) is provided.

## Methodology

| | |
| --- | --- |
| Sample preparation | C. glutamicum cells harboring sRNA and fluorescent plasmids were harvested by centrifugation at 13,200 rpm, washed with phosphate-buffered saline (PBS) and resuspended with the same buffer for flow cytometry analysis |
| Instrument | BD FACS LSRFortessa cell analyzer |
| Software | BD FACSDiva Software Version 6.2 |
| Cell population abundance | 10,000 cells were collected for analysis. |
| Gating strategy | Total population was taken for analysis. |

☒ Tick this box to confirm that a figure exemplifying the gating strategy is provided in the Supplementary Information.
